# Apigenin-induced lysosomal degradation of β-catenin in Wnt/β-catenin signaling

**DOI:** 10.1038/s41598-017-00409-z

**Published:** 2017-03-23

**Authors:** Chung-Ming Lin, Hsin-Han Chen, Chun-An Lin, Hui-Chung Wu, Jim Jinn-Chyuan Sheu, Hui-Jye Chen

**Affiliations:** 1 Department of Biotechnology, Ming-Chuan University, Taoyuan, 33348 Taiwan; 2 Division of Plastic and Reconstructive Surgery, Department of Surgery, China Medical University Hospital, Taichung, 40402 Taiwan; 3 Graduate Institute of Basic Medical Science, China Medical University, Taichung, 40402 Taiwan; 4 Institute of Biomedical Sciences, National Sun Yat-sen University, Kaohsiung, 80424 Taiwan; 5 Department of Nursing, Asia University, Taichung, 40354 Taiwan; 6 Graduate Institute of Biomedical Sciences, China Medical University, Taichung, 40402 Taiwan

## Abstract

The bioflavonoid apigenin has been shown to possess cancer-preventive and anti-cancer activities. In a drug screening, we found that apigenin can inhibit Wnt/β-catenin signaling, a pathway that participates in pivotal biological functions, which dis-regulation results in various human diseases including cancers. However, the underlying mechanism of apigenin in this pathway and its link to anti-cancer activities remain largely unknown. Here we showed that apigenin reduced the amount of total, cytoplasmic, and nuclear β-catenin, leading to the suppression in the β-catenin/TCF-mediated transcriptional activity, the expression of Wnt target genes, and cell proliferation of Wnt-stimulated P19 cells and Wnt-driven colorectal cancer cells. Western blotting and immunofluorescent staining analyses further revealed that apigenin could induce autophagy-mediated down-regulation of β-catenin in treated cells. Treatment with autophagy inhibitors wortmannin and chloroquine compromised this effect, substantiating the involvement of autophagy-lysosomal system on the degradation of β-catenin during Wnt signaling through inhibition of the AKT/mTOR signaling pathway. Our data not only pointed out a route for the inhibition of canonical Wnt signaling through the induction of autophagy-lysosomal degradation of key player β-catenin, but also suggested that apigenin or other treatments which can initiate this degradation event are potentially used for the therapy of Wnt-related diseases including cancers.

## Introduction

The natural flavone apigenin (4′,5,7-trihydroxyflavone) is abundant in fruits and vegetables. It is shown to be a bioactive flavonoid that possesses anti-inflammatory, antioxidant and anti-cancer activities^1^. Plant preparation that contains apigenin as traditional medicines for centuries in Europe is routinely used for the therapy of asthma, insomnia, neuralgia, shingles, Parkinson’s disease, and degeneration-related diseases^2^. Epidemiological investigation has shown that food stuff rich in flavones is related to a reduced risk of several cancers, especially cancers of skin, breast, prostate, digestive tract, and certain hematological malignancies^1^. Apigenin has been shown to interfere with the process of carcinogenesis and is regarded as a cancer-chemo-preventive agent. Besides, apigenin can inhibit tumor growth, invasion, and metastasis^3^.

Wnts are a group of secreted lipoglycoproteins that function as signaling molecules to regulate embryonic development at different stages and participate in adult tissue homeostasis^4, 5^. Dis-regulation in Wnt signaling causes a wide variety of human diseases such as leukemia, tetra-amelia, schizophrenia, kidney damage, bone morbidity, pulmonary fibrosis, and different kinds of cancers^6^. In the condition of without Wnt, members of the Wnt signaling pathway such as Axin, adenomatous polyposis coli (APC), glycogen synthase kinase 3β (GSK3β), casein kinase 1α (CK1α), microtubule actin crosslinking factor 1 (MACF1)^7^ and beta-catenin (CTNNB1) form a protein complex termed the “β-catenin destruction complex” or “Axin complex” in the cytoplasm. In this complex, β-catenin will be phosphorylated by GSK3β and CK1α on serines 33, 37, 45 and threonine 41 and subsequently be tagged with polyubiquitin before its destruction by the 26S proteasome degradation system. In the presence of Wnt, Wnt binds to its membranous receptor frizzled and co-receptor low-density lipoprotein receptor-related protein 5/6 (LRP5/6). Dishevelled (DVL), another member of Wnt signaling, will be phosphorylated and recruited to the cell membrane by binding to the receptor Frizzled. Thereafter the Axin complex will be translocated from the cytoplasm to the cell membrane with the help of MACF1^7^ and bind to phosphorylated LRP5/6 through Axin, and finally Axin will be degraded. β-catenin will be released, accumulated in the cytoplasm, move into the nucleus, bind to T-cell factor/lymphoid enhancer factor (TCF/LEF), and then activate the expression of Wnt target genes, such as c-Myc, cyclin D1 and Axin2^6, 8^.

With more than 1.3 million of people diagnosed each year, colorectal cancer (CRC) was among the most frequent cancers and was also one of the top cause of cancer-related death^9, 10^. Major causes for committing colorectal cancers include eating processed meat and red meat, smoking and drinking, obesity, a history of inflammatory Bowel diseases, and genetic variations that contribute to the inherited CRC, familial adenomatous polyposis (FAP), and hereditary nonpolyposis colorectal cancer (HNPCC)^11–13^. Traditional treatments for CRC are surgery, chemotherapy, radiotherapy, and targeting therapies^14^. Recently, a flurry in the progress of screening and prevention such as the developments in genomic analysis and biomarker, and the advancement in other non-traditional therapies such as immunotherapy and nutritional supplement therapy, has greatly reduced the mortality rates^14^. However, patients with an advanced and metastatic CRC are still hard to tackle with, suggesting that there is an urgent need to look for novel ways for the therapy of the disease.

Autophagy is a self-eating mechanism to recycle damaged proteins and organelles through the lysosomal degradation system^15, 16^, and thus autophagy can maintain cellular homoeostasis. Autophagy can also be induced by certain environmental stresses such as nutrient deficiency, oxygen deprivation, and cytotoxic agents^17^. There are at least two ways of autophagy-mediated degradation: one is the degradation of specific cellular components and invading micro-organisms, the other is the non-specific bulk degradation of cytoplasm. The detailed mechanisms underlying these specificities largely remain to be determined^17^. The process is initiated by the sequestration of intracellular candidate components into the small membrane structures called phagophores which then develop into the double–membrane vesicular structures termed autophagosomes, finally fusing with lysosomes to become autolysosomes and initiate the degradation. During the formation of autophagosomes, the mammalian homologues of yeast autophagy related protein Atg8, the microtubule-associated protein LC3, is processed by another autophagy related protein Atg4 to produce the active cytosolic LC3-I, which in turn activated by Atg3 to become the membrane-bound form, LC3-II. LC3-II then binds tightly to preautophagosomal, autophagosomal and autolysosomal membranes with its bound lipid moiety^18^. On the route to autophagy, LC3 binds to the autophagy adaptor sequestosome 1 (SQSTM1/p62) to facilitate the autophagic flux^19^. At the end of autophagy, cargos, LC3 and SQSTM1/p62 will eventually be degraded in the autolysosome.

In this paper, we found that the bioflavonoid apigenin can down-regulate β-catenin through activation of the autophagy-lysosomal degradation system by inhibiting the AKT/mTOR signaling pathway in Wnt-stimulated cells and Wnt-driven colorectal cancer cells, contributing to the suppression in Wnt signaling and thus the inhibition of cancer cell proliferation. These results suggest the potential application of harmless apigenin for the therapy of colorectal cancer as well as Wnt-related diseases, and also provide a novel therapeutic target for rational drug design.

## Results

### Apigenin inhibits Wnt/β-catenin signaling

**Figure 1:**
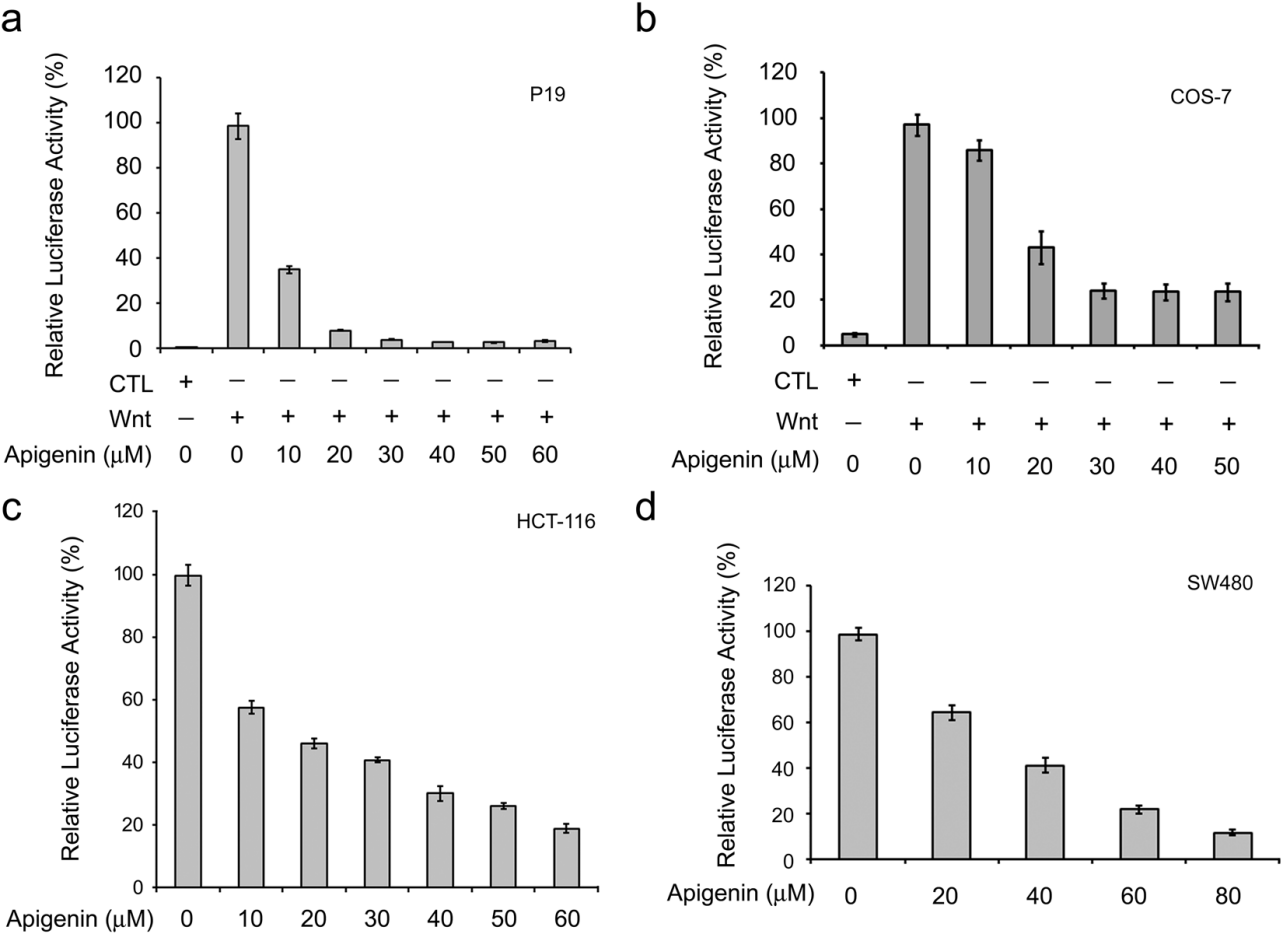
Apigenin inhibits Wnt signaling. (**a**) Apigenin inhibits Wnt signaling of Wnt-stimulated P19 cells. Cells were transfected with the Wnt reporter pGL3-OT and the normalization reporter pTK-Renilla (dual reporters) and treated with control-conditioned medium (CTL), Wnt-3a-conditioned medium (Wnt), or different concentrations (10~60 μM) of apigenin in Wnt-3a-conditioned medium for 16 h, and assayed for dual luciferase activities. (**b**) Apigenin inhibits Wnt signaling of Wnt-stimulated COS-7 cells. Reporter-transfected cells were treated with control-conditioned medium, Wnt-3a-conditioned medium, or different concentrations (10~50 μM) of apigenin in Wnt-3a-conditioned medium for 20 h, and assayed for dual luciferase activities. Each treatment is in three replicates and data were mean ± S.D. The dual luciferase activity of Wnt-stimulated cells was set as 100% and the relative activity of other treatment was calculated accordingly. (**c**) Apigenin inhibits intrinsic Wnt signaling in HCT-116 cells. Cells were transfected with dual reporters, treated with different concentrations (0~60 μM) of apigenin for 22 h, and then assayed for dual luciferase activities. (**d**) Apigenin inhibits intrinsic Wnt signaling in SW480 cells. Reporter-transfected cells were treated with different concentrations (0~80 μM) of apigenin for 22 h, and assayed for dual luciferase activities. Each treatment is in three replicates and data were mean ± S.D. The luciferase activity of treatment without any drug was set as 100%, and the relative activity of other treatment was calculated accordingly.

Wnt signaling is dis-regulated in many human diseases^20^ and drugs that can block aberrant Wnt signaling would be promising for the therapy of these diseases. In order to look for potential drugs that inhibit Wnt/β-catenin signaling, we have set up a drug screening platform based on the β-catenin/TCF-mediated transcriptional activity assay^21^ and have successfully picked up several candidates that can inhibit Wnt signaling from natural compounds, modified compounds, and Chinese herbs, and one of them is apigenin. Apigenin is a flavonoid rich in parsley, celery, and chamomile and has been shown to possess cancer-preventive and anti-cancer activities^22^. To confirm the effects of apigenin on the Wnt/β-catenin signal transduction pathway, we treated Wnt-reporter-transfected P19 cells in control-conditioned medium, Wnt-3a-conditioned medium, or with apigenin in Wnt-3a-conditioned medium, and analyzed the Wnt-induced β-catenin/TCF-mediated luciferase activity. P19 cells are pluripotent embryonic carcinoma cells with stem cell properties that can be differentiated into three germ layers upon stimulation^23^. Our previous data have shown that P19 cells have good Wnt response and are suitable for the study of Wnt signaling^21^. As shown in Fig. 1a, apigenin inhibited the β-catenin/TCF-mediated transcriptional activity in a concentration-dependent manner, with more than 60% of inhibition at concentrations higher than 10 μM of apigenin. Apigenin also dose-dependently decreased the β-catenin/TCF-mediated luciferase activity in Wnt-stimulated COS-7 cells (Fig. 1b). As colorectal cancer cell lines such as HCT-116 and SW480 cells have modest to high intrinsic Wnt activity^24^, we explored the effects of apigenin on this activity. As shown in Fig. 1c,d, apigenin treatment suppressed the β-catenin/TCF-mediated luciferase activity in Wnt-driven HCT-116 and SW480 colorectal cancer cells. Therefore, these data demonstrated that apigenin is able to inhibit Wnt/β-catenin signaling.

### Apigenin inhibits the expression of Wnt target genes

**Figure 2:**
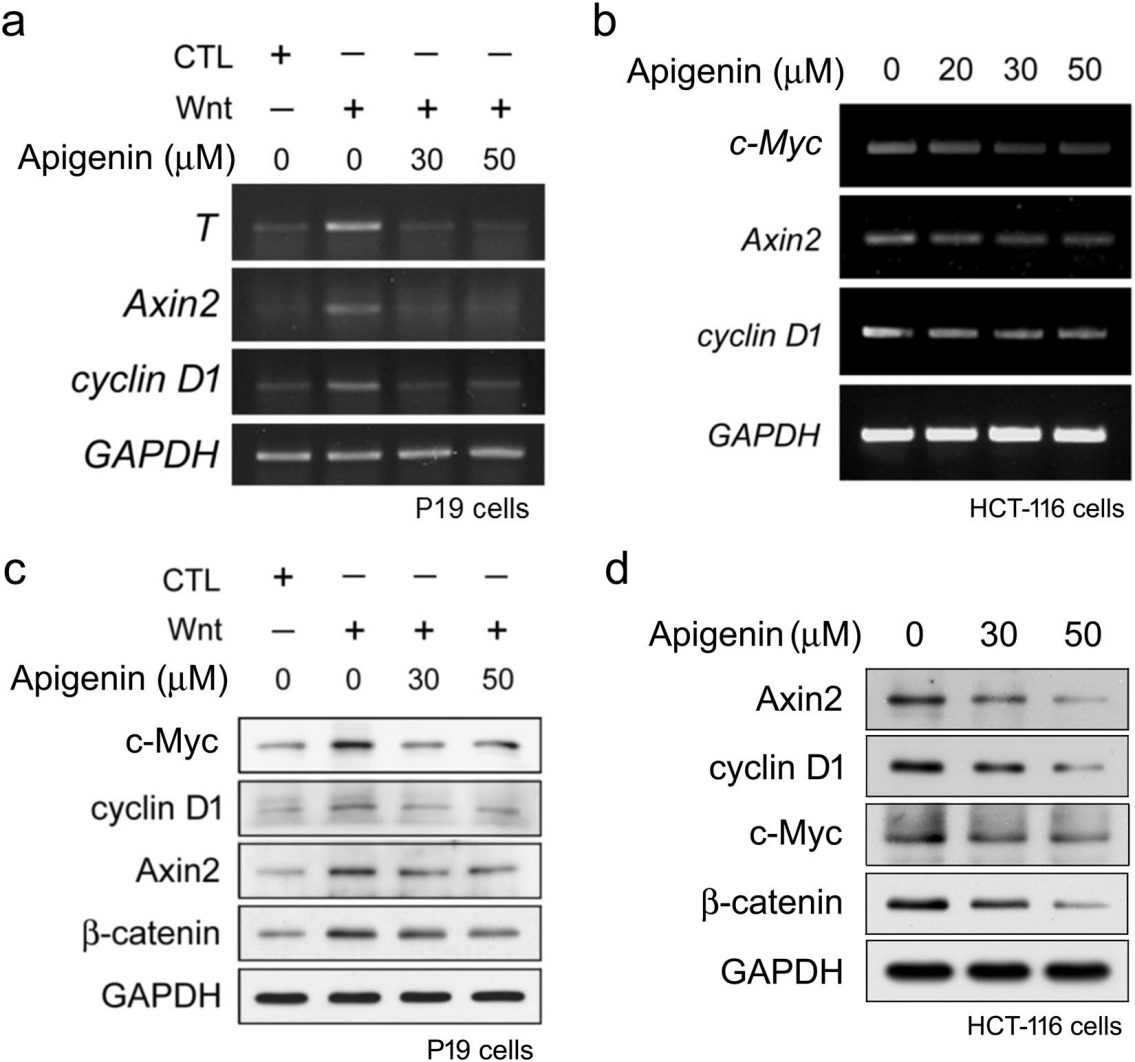
Apigenin suppresses the expression of Wnt target genes. (**a**) Apigenin inhibits the mRNA expression of Wnt target genes in Wnt-stimulated P19 cells. Cells were treated with control-conditioned medium, Wnt-3a-conditioned medium, or different concentrations (30, 50 μM) of apigenin in Wnt-3a-conditioned medium for 16 h, and then cells were collected for RT-PCR analyses using gene-specific primers for *T*, *Axin2*, *cyclin D1*, and *GAPDH* (used as the internal control) as described in methods. (**b**) Apigenin represses the mRNA expression of Wnt target genes in HCT-116 cells. Cells were treated with different concentrations (0, 20, 30, 50 μM) of apigenin for 22 h and total RNAs were isolated. cDNAs were then synthesized and used for amplification of the specified genes using gene-specific primers for *c*-*Myc*, *Axin2*, *cyclin D1*, and *GAPDH*. (**c**) Apigenin inhibits the protein expression of Wnt target genes in Wnt-stimulated P19 cells. Cells were treated with control-conditioned medium, Wnt-3a-conditioned medium, or different concentrations (30, 50 μM) of apigenin in Wnt-3a-conditioned medium for 16 h, and then cells were collected for Western blotting analyses with anti-c-Myc, anti-cyclin D1, anti-Axin2, anti-beta-catenin, and anti-GAPDH (as the loading control). (**d**) Apigenin inhibits the protein expression of Wnt target genes in HCT-116 colorectal cancer cells. Cells were treated with different concentrations (0, 30, 50 μM) of apigenin for 22 h and total cell lysates were isolated for Western blotting analyses with anti-Axin2, anti-cyclin D1, anti-c-Myc, anti-β-catenin, and anti-GAPDH.

Given that apigenin inhibits Wnt/β-catenin signaling, we wonder whether apigenin also decreases the expression of Wnt-responsive genes such as *cyclin D1*, *c*-*Myc*, *T* (*brachyury*), and *Axin2* (*conductin*). RT-PCR analyses showed that Wnt-3a treatment induced the mRNA expression of Wnt target genes including *T*, *Axin2*, and *cyclin D1* in Wnt-stimulated P19 cells. Treatment with apigenin decreased the expression of these genes (Fig. 2a). Apigenin treatment also decreased the mRNA expression of Wnt target genes including *c*-*Myc*, *Axin2*, and *cyclin D1* in Wnt-active HCT-116 cells (Fig. 2b). These results were further confirmed by quantitative real-time PCR (qRT-PCR) analyses (Supplementary Figs S1 and S2). We then addressed whether apigenin also inhibits the protein expression of Wnt target genes. Wnt treatment induced the protein expression of Wnt target genes such as c-Myc, cyclin D1, and Axin2 in P19 cells. After treatment with 30 μM and 50 μM of apigenin, the protein levels of these Wnt target genes were greatly reduced (Fig. 2c). Furthermore, apigenin treatment suppressed the expression in the protein levels of Wnt target genes in HCT-116 colorectal cancer cells (Fig. 2d). These data indicated that apigenin is able to inhibit the target gene expression of Wnt/β-catenin signaling.

### Apigenin inhibits cell proliferation of Wnt-stimulated P19 cells and Wnt-driven colorectal cancer cells

**Figure 3:**
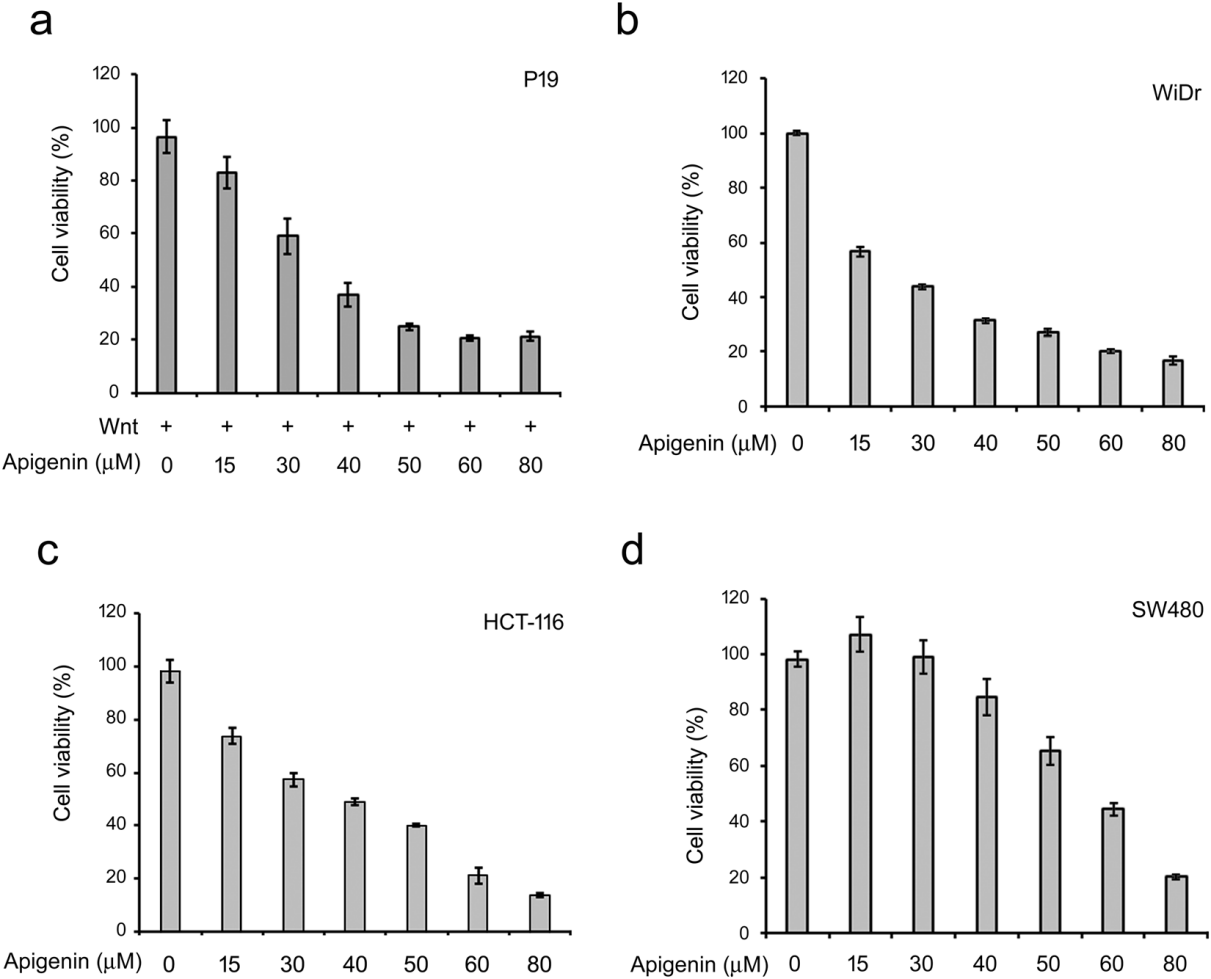
Apigenin decreases the cell survival of Wnt-stimulated P19 cells and Wnt-driven colorectal cancer cells. (**a**) Apigenin suppresses the survival of Wnt-stimulated P19 cells. Cells were treated with different concentrations (0~80 μM) of apigenin in Wnt-3a-conditioned medium (Wnt, +) for 4 days and then subjected to MTT assay. The calculated IC50 of apigenin for Wnt-stimulated P19 cells is about 38.15 ± 1.32 μM. (**b**–**d**) Apigenin suppresses the survival of WiDr, HCT-116 and SW480 cells. Cells were treated with different concentrations (0~80 μM) of apigenin and then subjected to MTT assay. The calculated IC50 of apigenin for WiDr, HCT-116 and SW480 cells is about 29.69 ± 0.15, 39.67 ± 0.99, and 61.38 ± 2.29 μM respectively. Each treatment is in five replicates and data were mean ± S.D.

As Wnt signaling is important for cell proliferation^5^ and apigenin can inhibit Wnt/β-catenin signaling, we assessed the effects of apigenin on cell growth of Wnt-stimulated P19 cells and Wnt-driven colorectal cancer cells including HCT-116, SW480, and WiDr cells. As shown in Fig. 3a, apigenin inhibited cell growth of Wnt-stimulated P19 cells in a concentration-dependent manner by MTT assay, with the calculated IC50 to be 38.15 ± 1.32 μM. More than 60% of inhibition in cell growth was observed at concentrations higher than 40 μM.

More than 80% of CRC are initiated by mutations in adenomatous polyposis coli (APC) or β-catenin genes^14, 25^. Both SW480 and WiDr cells have APC mutations that result in the accumulation of β-catenin, while HCT-116 cells harbor β-catenin mutation that prevents its ubiquitin-dependent proteolysis by the proteasomal system^26^. These cells were termed Wnt-driven cells due to the stabilization and accumulation of β-catenin within the cells, and hence the constitutive transduction of β-catenin-mediated downstream Wnt signaling. As previously described, apigenin is able to inhibit the Wnt/β-catenin signaling cascade (Figs 1 and 2) and inhibit the cell survival of Wnt-stimulated P19 cells (Fig. 3a), we wanted to know whether apigenin also can suppress cell growth of Wnt-driven colorectal cancer cells. To address this, cells were treated with different concentrations of apigenin and cell viability was examined by MTT assay. As shown in Fig. 3b–d, apigenin suppressed cell growth of WiDr, HCT-116, and SW480 cells concentration-dependently, with the IC50 to be 29.69 ± 0.15 μM, 39.67 ± 0.99 μM, and 61.38 ± 2.29 μM respectively. To further examine the effect of apigenin on the progression of cell cycle, we treated Wnt-activated P19 cells and Wnt-driven colorectal cancer cells with apigenin and the cell cycle distribution was revealed by flow cytometry. As shown in Supplementary Figs S3 and S4, apigenin treatment induced cell cycle arrest at G2/M phase in Wnt-stimulated P19 cells and Wnt-driven HCT-116, SW480, and WiDr colorectal cancer cells. Taken together, these results showed that apigenin can inhibit cell cycle progression and thus cell proliferation no matter in Wnt-stimulated cells or Wnt-driven colorectal cancer cells.

### Apigenin acts downstream of LRP5 and Dishevelled in the Wnt/β-catenin signaling cascade

**Figure 4:**
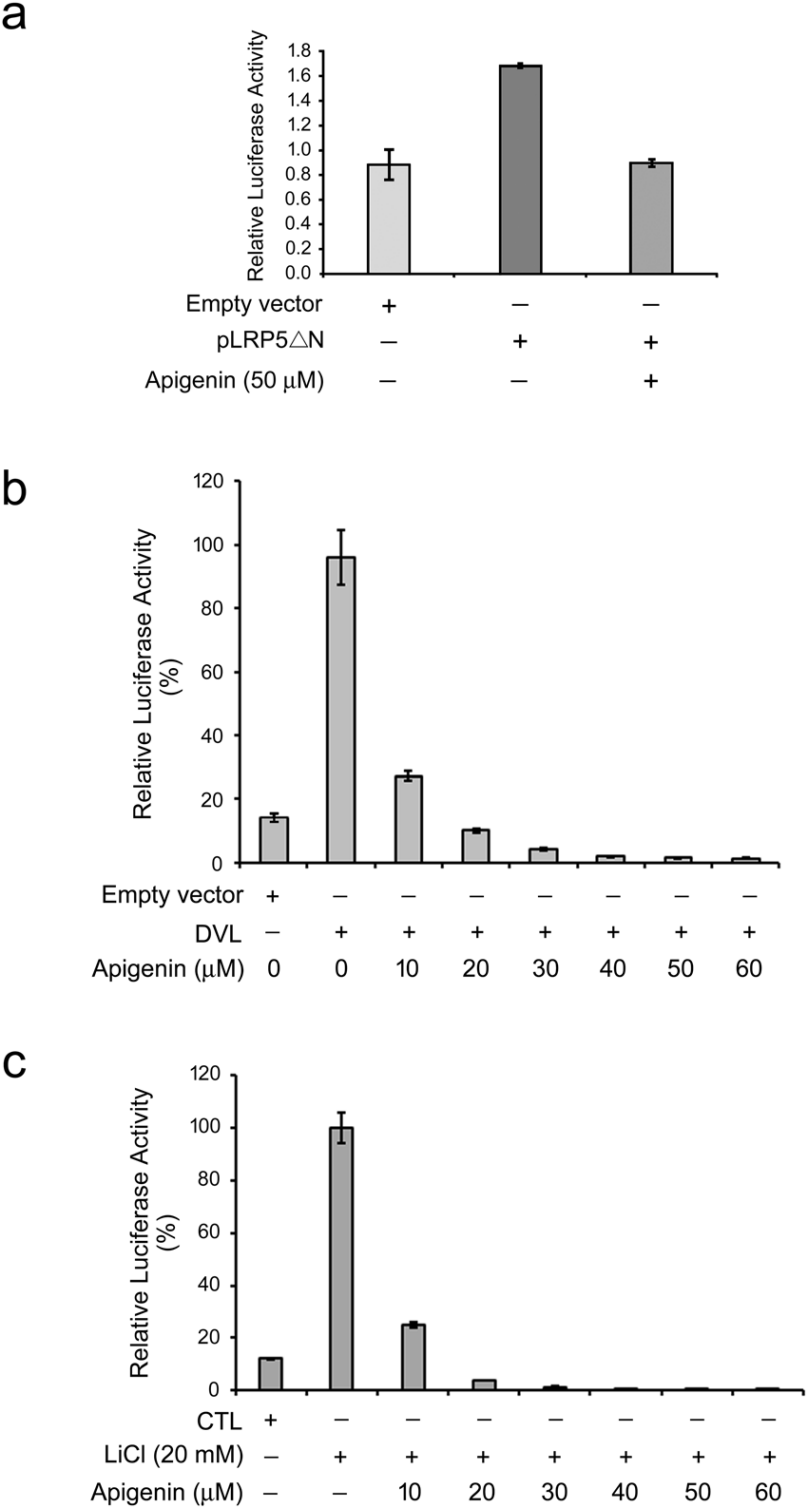
Apigenin acts on GSK3β *per se* or downstream of GSK3β in the Wnt signaling pathway. (**a**) Apigenin acts on LRP5 or downstream of LRP5 in the Wnt/β-catenin signaling pathway. P19 cells were transfected with two reporter plasmids together with pLRP5ΔN (grey bar and heavy grey bar), or empty vector (light grey bar), then treated with 50 μM of apigenin, and assayed for dual luciferase activities. The luciferase activity from the expression of empty vector alone was set as 1.0, and the relative activity of other treatment was calculated accordingly. (**b**) Apigenin acts on DVL or downstream of DVL in the Wnt signal transduction pathway. P19 cells were transfected with two reporter plasmids together with DVL2 plasmid, or empty vector, then treated with different concentrations (0~60 μM) of apigenin, and assayed for dual luciferase activities. The luciferase activity from the expression of DVL alone was set as 100%, and the relative activity of other treatment was calculated accordingly. (**c**) Apigenin acts on GSK3β or downstream of GSK3β in the Wnt signaling pathway. P19 cells were transfected with two reporter plasmids, incubated in culture medium (CTL) or treated with 20 mM of LiCl as well as different concentrations (0~60 μM) of apigenin in culture medium, and then assayed for dual luciferase activities. The luciferase activity of LiCl treatment alone was set as 100%, and the relative activity of other treatment was calculated accordingly.

Since apigenin inhibited Wnt/β-catenin signaling and cell proliferation, we began to look for the cellular targets of apigenin in the Wnt/β-catenin signaling pathway. We first checked whether apigenin acts upstream or downstream of LRP5/6. To this end, P19 cells were transfected with a Wnt reporter, a normalization vector, the constitutive active mutant of LRP5, pLRP5ΔN, and treated with 50 μM of apigenin for dual reporter activity assay. Over-expression of LRP5ΔN will cause the constitutive activation of Wnt signaling downstream of LRP5^27^. Ectopic expression of LRP5ΔN indeed induced the β-catenin/TCF-mediated luciferase activity and treatment with apigenin decreased this activity (Fig. 4a), suggesting that apigenin functions on LRP5 *per se* or downstream of LRP5. We then examined if apigenin acts upstream or downstream of Dishevelled (DVL). To address this, P19 cells were transfected with dual reporters, the 3X-Flag-tagged DVL2^28^ and treated with different concentrations (0~60 μM) of apigenin. Cell lysates were then obtained for dual luciferase activity assay. As shown in Fig. 4b, expression of DVL elicited a dramatic β-catenin/TCF-mediated transcriptional activity in P19 cells. Treatment with apigenin inhibited the DVL-elicited β-catenin/TCF-mediated transcriptional activity in a concentration-dependent manner. More than 70% of inhibition of this activity was observed at concentrations higher than 10 μM of apigenin, and complete inhibition was at concentrations higher than 20 μM, suggesting that apigenin acts on DVL itself or some component downstream of DVL.

### Apigenin acts on β-catenin stabilization of the Wnt/β-catenin signaling pathway

As mentioned above, apigenin probably targets on downstream of DVL and LRP5 of the Wnt/β-catenin signaling cascade. We are then interested to know whether apigenin acts upstream or downstream of GSK3β. To this purpose, P19 cells were transfected with dual reporters, treated with the GSK3β inhibitor LiCl, and cell lysates were collected for dual luciferase activity assay. Treatment of cells with LiCl will result in the stabilization and accumulation of β-catenin in the cytoplasm, contributing to β-catenin-mediated nuclear signaling^29^. As compared to cells without any treatment, treatment with LiCl induced a strong β-catenin/TCF-mediated luciferase activity. Treatment with apigenin substantially suppressed the LiCl-triggered β-catenin/TCF-mediated transcriptional activity, with more than 75% of inhibition at concentrations higher than 10 μM (Fig. 4c). Complete inhibition was observed at concentrations higher than 20 μM of apigenin. This result suggested that apigenin acts on GSK3β *per se* or some components downstream of GSK3β.

**Figure 5:**
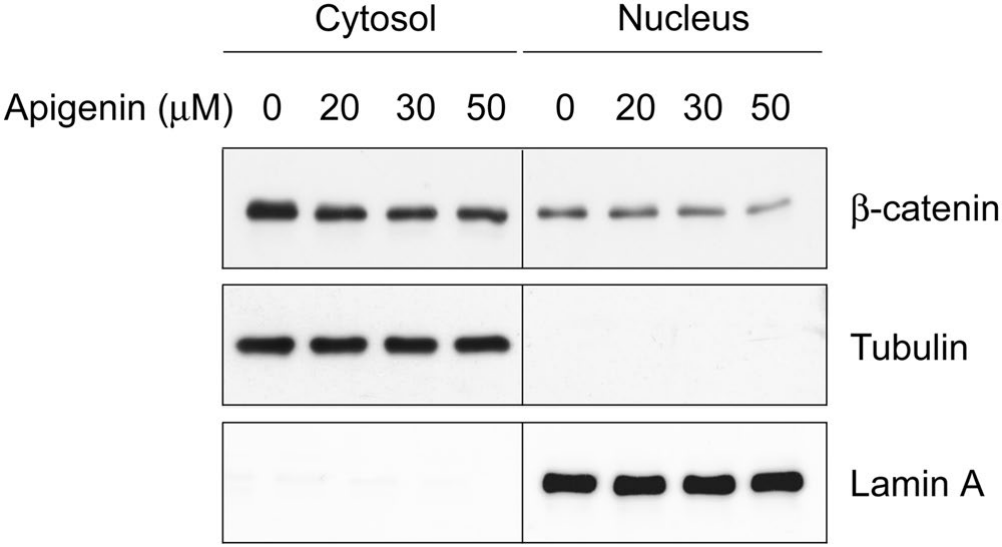
Apigenin decreases the protein levels of cytoplasmic and nuclear β-catenin. HCT-116 cells were treated with different concentrations of apigenin (0, 20, 30, 50 μM) for 22 h. Cells were collected for the isolation of cytosol and nuclear fractions as described in methods. Cytosolic and nuclear lysates were subjected to Western blotting analyses with anti-β-catenin, anti-tubulin, and anti-lamin A. Tubulin served as the cytosolic marker and lamin A served as the nuclear marker.

**Figure 6:**
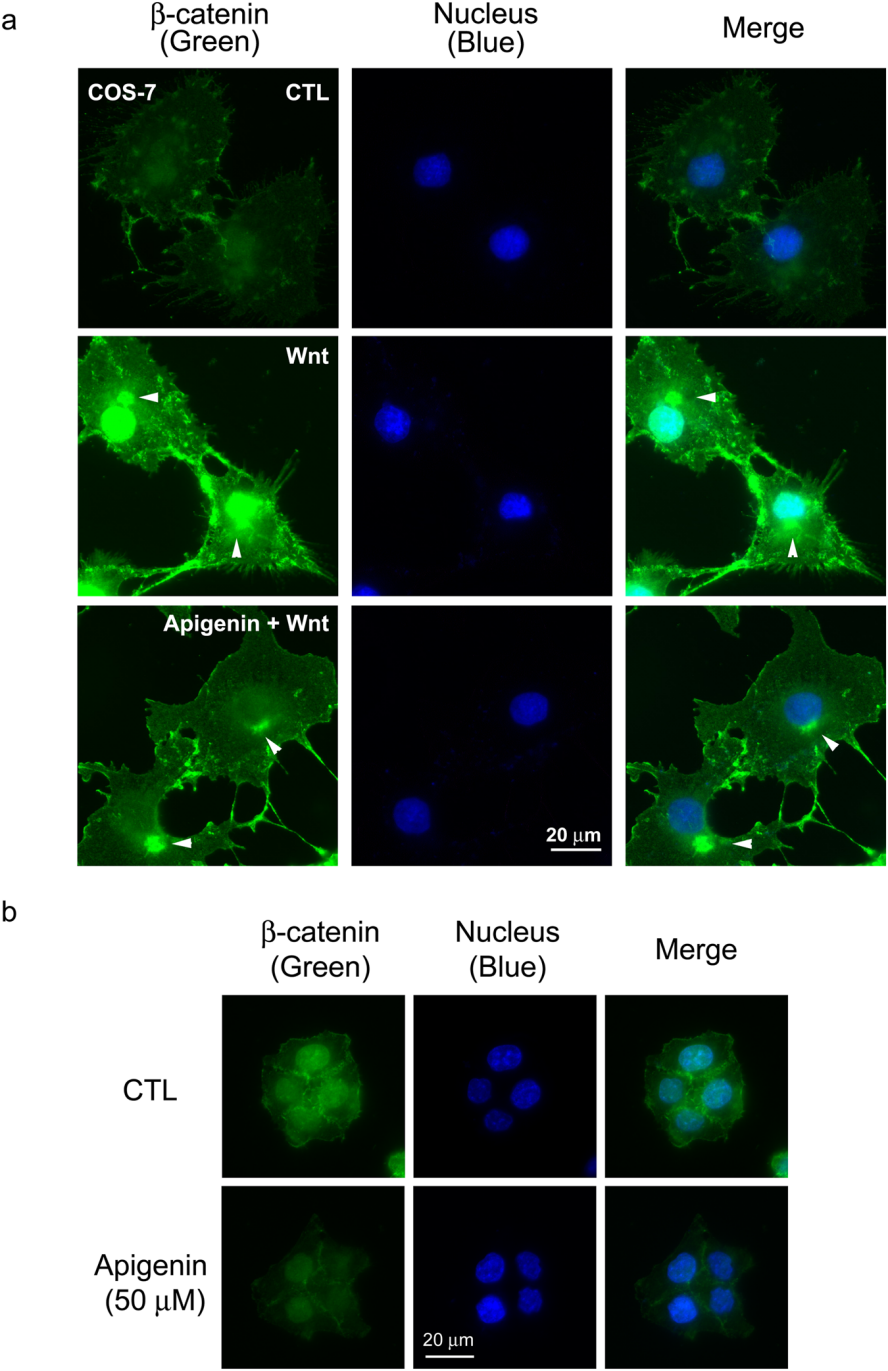
Apigenin reduces the levels of β-catenin in Wnt-stimulated COS-7 cells and HCT-116 cells by immunofluorescent staining. (**a**) Apigenin decreases the levels of β-catenin in Wnt-stimulated COS-7 cells. Cells were treated with control-conditioned medium (CTL), Wnt-3a-conditioned medium (Wnt), or 50 μM of apigenin in Wnt-3a-conditioned medium (Apigenin + Wnt) for 20 h, and cells were processed for immunofluorescent staining with β-catenin antibody (labeled in green). Cell nuclei were stained with DAPI (labeled in blue). Arrowheads showed the staining of β-catenin on microtubule-organizing center (MTOC). (**b**) Apigenin decreases the levels of β-catenin in HCT-116 cells. Cells were either incubated in culture medium (CTL) or treated with 50 μM of apigenin in culture medium for 22 h and processed for immunofluorescent staining with β-catenin antibody (labeled in green). Cell nuclei were stained with DAPI (labeled in blue).

We next examined the effects of apigenin on β-catenin stabilization. As shown in Fig. 2c (Panel 4), Wnt stimulation induced β-catenin stabilization and accumulation in P19 cells. Apigenin treatment decreased the protein levels of β-catenin in a concentration-dependent manner. Apigenin treatment also resulted in the reduction of β-catenin levels in HCT-116 cells and WiDr cells (Panel 4 in Fig. 2d and Panel 1 in Fig. 8c,d). It is well-known that, during Wnt signaling, β-catenin will be stabilized and accumulated in the cytoplasm and then translocates into the nucleus to activate the expression of Wnt-responsive genes. To know apigenin exerts its effects on which pool of β-catenin, we performed cell fractionation of apigenin-treated and non-treated cells. Cytosolic and nuclear fractions were obtained for Western blotting analyses. Apigenin treatment decreased the β-catenin levels both in the cytosol and nucleus (Fig. 5). We also examined the subcellular distribution of β-catenin in COS-7 cells incubated in control-conditioned medium, in Wnt-3a-conditioned medium, or with apigenin in Wnt-3a-conditioned medium by immunofluorescent staining. As compared to cells treated with control-conditioned medium, Wnt treatment increased the β-catenin levels both in the cytoplasm and nucleus, while apigenin treatment reduced the levels of β-catenin in both subcellular compartments of Wnt-stimulated COS-7 cells (Fig. 6a). The same phenomenon was observed in apigenin-treated HCT-116 cells, as compared to non-treated cells (Fig. 6b). These results of immunofluorescent staining confirmed the cell fractionation data. Therefore, apigenin can reduce the amount of total, cytoplasmic, and nuclear β-catenin in Wnt-challenged and Wnt-driven cells.

### Autophagy-lysosomal system is involved in apigenin-induced β-catenin degradation during Wnt signaling

**Figure 7:**
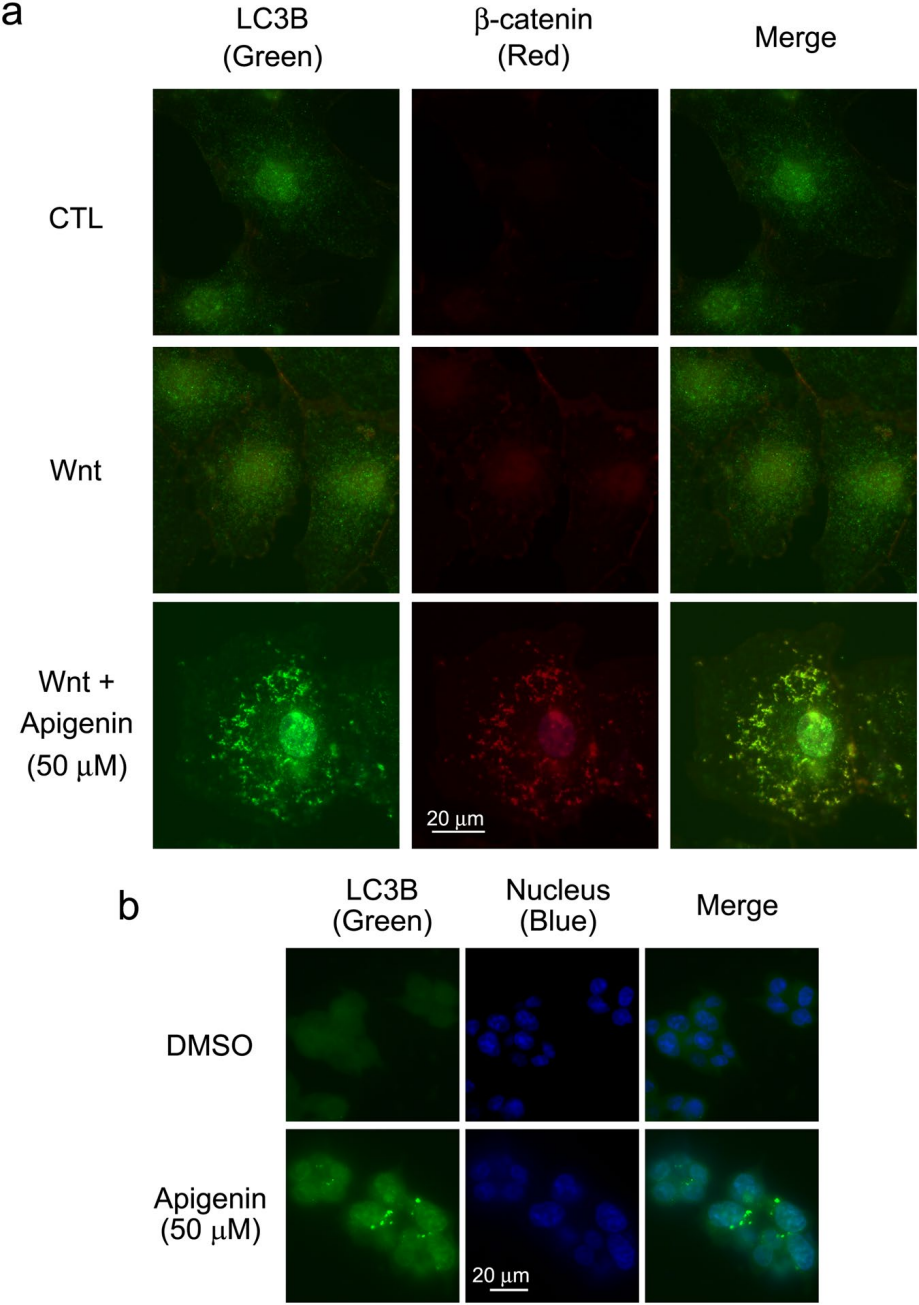
Apigenin induces the formation of autophagosomes in cells. (**a**) Apigenin induces the formation of autophagosomes in Wnt-stimulated COS-7 cells. COS-7 cells were treated with control-conditioned medium (CTL), Wnt-3a-conditioned medium (Wnt), or 50 μM of apigenin in Wnt-3a-conditioned medium (Wnt + 50 μM Apigenin) for 20 h, and cells were processed for immunofluorescent staining with LC3B antibody (labeled in green) and β-catenin antibody (labeled in red). (**b**) Apigenin induces the formation autophagosomes in Wnt-driven HCT-116 cells. Cells were treated with carrier reagent (DMSO) or 50 μM of apigenin for 22 h and processed for immunofluorescent staining with LC3B antibody (labeled in green). Cell nuclei were stained with DAPI (labeled in blue).

The aforementioned data indicated that apigenin treatment decreased the β-catenin levels in Wnt-challenged cells and Wnt-driven cells. We suspected that apigenin could interfere with the stabilization and accumulation of β-catenin in the canonical Wnt signal transduction pathway. There are three major protein proteolysis systems which include the ubiquitin-proteasome system, lysosomal degradation system, and calpain system in cells^30^. We ruled out the possibility that the ubiquitin-proteasome system is involved in this process. The colorectal cancer cell line HCT-116 harbors a β-catenin mutation^26^ that prevents its degradation by the proteasomal system. However, our previous data showed that apigenin still decreased the β-catenin levels and β-catenin-mediated Wnt signaling in HCT-116 cells (Figs 1c and 2d), suggesting that other degradation system is responsible for the apigenin-induced β-catenin degradation during Wnt signaling. We therefore examined the participation of autophagy-lysosomal degradation system in this process. COS-7 cells were treated with apigenin in the presence of Wnt and the subcellular distribution of β-catenin and the autophagy marker LC3B^31^ in cells were checked by immunofluorescent staining. As compared to cells treated with control-conditioned medium, treatment of cells with Wnt increased the amount of β-catenin. Apigenin treatment substantially induced the aggregation of both LC3B and β-catenin in cells. Interestingly, the punctuate staining of LC3B co-localized with the staining of β-catenin (Panel 3, Fig. 7a). Apigenin treatment also induced the aggregation of LC3B in HCT-116 cells (Fig. 7b). These data suggested the involvement of autophagy in apigenin-induced β-catenin down-regulation during Wnt signaling.

**Figure 8:**
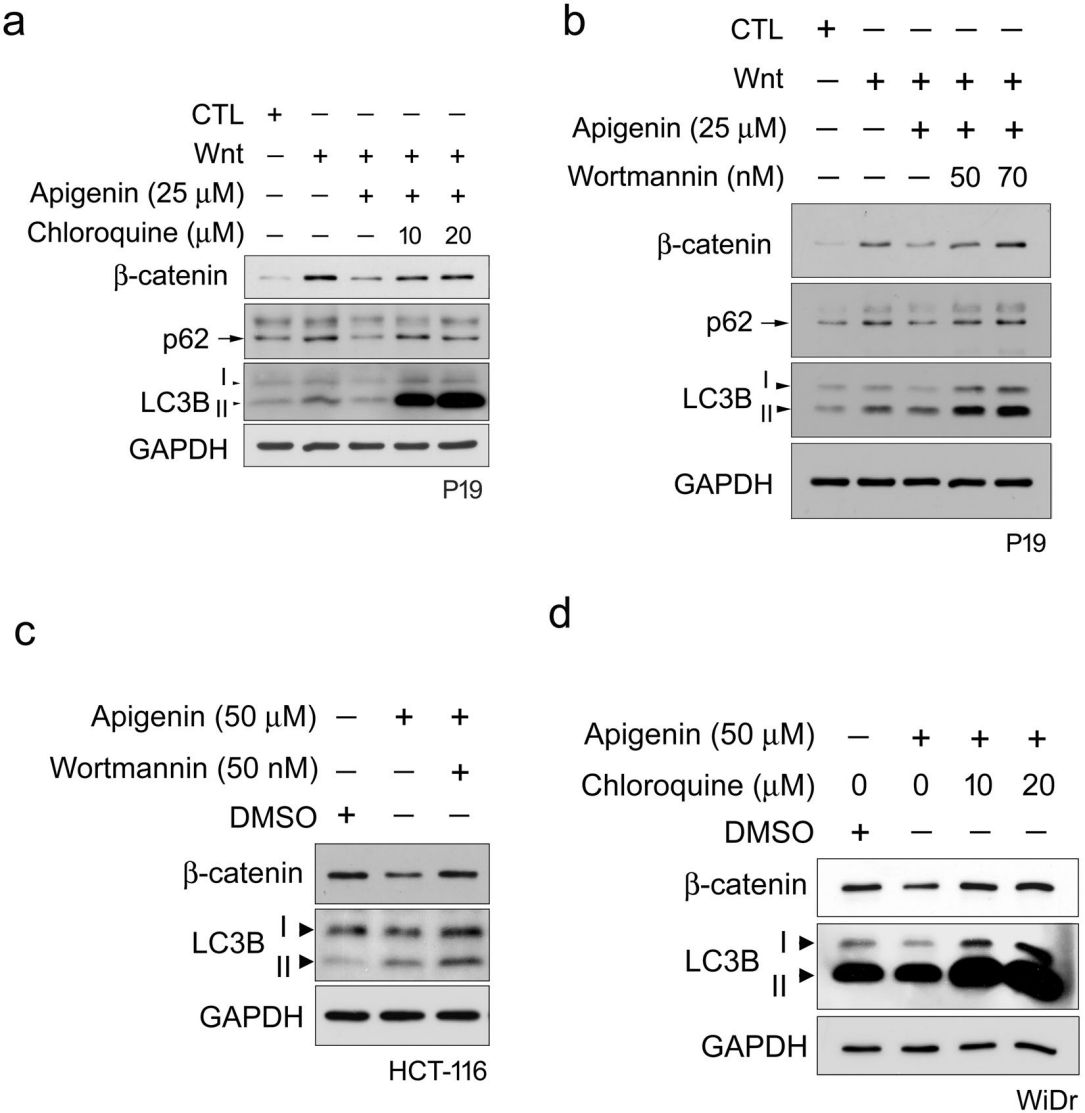
Autophagy-lysosomal degradation system is involved in apigenin-induced down-regulation of β-catenin during Wnt signaling. Autophagy inhibitor chloroquine (**a**) and wortmannin (**b**) inhibit the apigenin-induced degradation of β-catenin upon Wnt stimulation. P19 cells were incubated with control-conditioned medium (CTL), Wnt-3a-conditioned medium (Wnt) or treated with 25 μM of apigenin in Wnt-3a-conditioned medium for 16 h. For autophagy inhibitor treatment, cells were pre-treated with chloroquine (**a**) and wortmannin (**b**) for 2 h. Cells were then treated with 25 μM of apigenin and different concentrations of (**a**) chloroquine (10 and 20 μM) or (**b**) wortmannin (50 and 70 nM) in Wnt-3a-conditioned medium for 16 h. All cells were collected for Western blotting analyses with β-catenin, p62, LC3B, and GAPDH antibodies. GAPDH served as a loading control. (**c**) Wortmannin suppresses the apigenin-induced degradation of β-catenin in Wnt-driven HCT-116 cells. Cells were incubated with carrier reagent (DMSO) or 50 μM of apigenin for 22 h. For treatment with autophagy inhibitor, cells were pre-treated with 50 nM of wortmannin for 2 h, and then treated with 50 μM of apigenin and 50 nM of wortmannin for 22 h. All cells were collected for Western blotting analyses with β-catenin, LC3B, and GAPDH antibodies. GAPDH served as a loading control. (**d**) Chloroquine represses the apigenin-induced degradation of β-catenin in Wnt-driven WiDr cells. Cells were incubated with carrier reagent (DMSO) or 50 μM of apigenin for 22 h. For treatment with autophagy inhibitor, cells were pre-treated with the inhibitor for 2 h. Cells were then treated with 50 μM of apigenin together with different concentrations (10 and 20 μM) of chloroquine for 22 h. All cells were collected for Western blotting analyses with β-catenin, LC3B, and GAPDH antibodies. GAPDH served as a loading control.

To further check the role of autophagy-mediated lysosomal degradation system in this process, we treated P19 cells with apigenin and autophagy inhibitor chloroquine in the presence of Wnt for Western blotting analyses of β-catenin and two marker proteins that involve in autophagy, p62 and LC3B^32^. Chloroquine can inhibit the acidification of lysosome, thus prohibiting the lysosomal protein degradation^33^. As shown in Fig. 8a, Wnt treatment induced the accumulation of β-catenin, which was suppressed by the treatment with apigenin. Chloroquine treatment regained the accumulation of β-catenin, no matter at 10 μM or 20 μM of drug concentration. We also treated P19 cells with apigenin and another autophagy inhibitor wortmannin in the presence of Wnt for Western blotting analyses. Wortmannin is an inhibitor to PI3K, a kinase that is required for autophagy. Treatment with wortmannin would inhibit the autophagic sequestration^34^. As shown in Fig. 8b, Wnt treatment induced the accumulation of β-catenin, which was down-regulated after treatment with apigenin, and this effect was compromised by treatment with wortmannin. Treatment of HCT-116 and WiDr colorectal cancer cells with apigenin and wortmannin or apigenin and chloroquine respectively also rescued the apigenin-elicited β-catenin degradation in these cells (Fig. 8c,d). These data strongly indicated the involvement of autophagy-mediated lysosomal degradation system in the apigenin-elicited down-regulation of β-catenin during Wnt signaling.

### Apigenin induces autolysosomal degradation of β-catenin through inhibition of the AKT/mTOR signaling pathway during Wnt signaling

**Figure 9:**
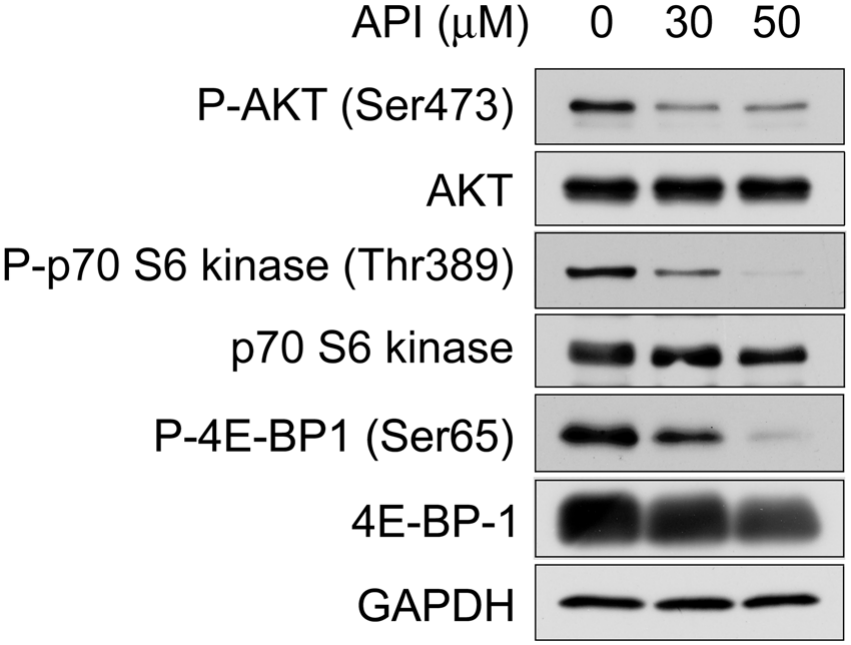
Apigenin-mediated down-regulation of β-catenin is through inhibition of the AKT/mTOR signaling pathway. HCT-116 cells were treated with different concentrations of apigenin (0, 30 and 50 μM) for 20 h, and the protein expression of phospho-AKT (Ser473), AKT, phospho-p70 S6 kinase (Thr389), p70 S6 kinase, phospho-4E-BP1 (Ser65), and 4E-BP1 was examined by Western blotting analyses with the antibodies respectively. GAPDH serves as a loading control.

Previous data have shown that autophagy-mediated lysosomal degradation system is involved in the apigenin-elicited down-regulation of β-catenin during Wnt signaling. It is known that the AKT/mammalian target of rapamycin (mTOR) signaling pathway regulates autophagy^35–37^. We therefore investigated the role of the AKT/mTOR signaling pathway in apigenin-induced autolysosomal degradation of β-catenin. To address this, HCT-116 cells were incubated with different concentrations of apigenin and Western blotting analyses were employed to examine the expression of signaling proteins including AKT, phosphorylated AKT (Ser473), p70 S6 kinase, phosphorylated p70 S6 kinase (Thr389), 4E-BP-1, and phosphorylated 4E-BP-1 (Ser65). As shown in Fig. 9, apigenin treatment decreased the active phosphorylated form of AKT, while the total levels of AKT were not affected, suggesting the involvement of AKT in this process. Besides, apigenin exposure reduced the levels of phosphorylated p70 S6 kinase and phosphorylated 4E-BP1, two mTOR downstream substrates, although that total levels of both proteins were slightly reduced when higher concentration of apigenin was used (Fig. 9). These data indicated that autolysosomal degradation of β-catenin by apigenin is through the repression of AKT/mTOR signaling pathway during Wnt signaling.

## Discussion

The Wnt/β-catenin signaling pathway plays important roles in embryonic development, adult tissue homeostasis, and stem cell maintenance. In the absence of Wnt ligand, the key player β-catenin is phosphorylated, poly-ubiquitinated, and then degraded by the 26S proteasome. In the presence of Wnt ligand, this pathway is activated by the cytoplasmic stabilization and accumulation of β-catenin, which in turn mobilizes into the nucleus to associate with TCF/LEF family of proteins to activate the expression of Wnt-responsive genes such as cyclin D1, c-Myc and Axin2. Mutations in members of this pathway such as APC, Axin, and β-catenin result in the accumulation of β-catenin, leading to continuous cell proliferation that eventually causes cancers^20^. For example, mutation in APC gene that defects in β-catenin degradation can ultimately result in the formation of colorectal cancers^38^. Thus, any strategies that can down-regulate the deregulated β-catenin will contribute to the therapy of the diseases. The natural flavonoid apigenin has been shown to disturb the process of carcinogenesis, inhibit tumor cell growth, make cancer cells sensitive to apoptosis, and hinder the development of metastasis^22^. Moreover its low toxicity to normal cells and strong activities to cancerous cells^39^ make apigenin appear to be an ideal agent for chemoprevention and cancer therapy. We are therefore interested to know whether the effect of apigenin on tumorigenesis, especially in colorectum, is through the inhibition of canonical Wnt signaling. In this paper, we discovered that the phytochemical apigenin is able to suppress Wnt/β-catenin signaling (Fig. 1), the expression of Wnt target genes (Fig. 2; Supplementary Figs S1 and S2), cell cycle progression (Supplementary Figs S3 and S4), and consequently un-controlled cell proliferation (Fig. 3) in Wnt-challenged cells and Wnt-active colorectal cancer cells. These results suggest that apigenin could have a potential for the therapy of Wnt-related diseases through the suppression of aberrant Wnt signaling.

To know the action mechanism and molecular target of apigenin in the Wnt/β-catenin signaling pathway, three approaches were used. We first expressed the constitutive-active version of LRP5, LRP5ΔN, and DVL respectively in apigenin-treated cells for dual luciferase activity assay and the results showed that apigenin inhibited β-catenin/TCF-mediated luciferase activities in LRP5ΔN- and DVL-overexpressed cells (Fig. 4a,b), indicating that apigenin works on LRP5 and DVL *per se* or some Wnt signaling components downstream of them. With Wnt, DVL will be phosphorylated by CK1δ and CK1ε and then participate in transducing downstream Wnt signaling. It is reported that phosphorylation of DVL by CKII can be inhibited by apigenin^40^. We reasoned that if apigenin can specifically inhibit CKII activity and hence repress the phosphorylation of DVL, it would not interfere with the LiCl-induced downstream Wnt signaling in our system. Therefore we treated cells with apigenin and LiCl for dual luciferase activity assay. To our surprise, apigenin inhibited LiCl-elicited β-catenin/TCF transcriptional activity (Fig. 4c), implying that the inhibition of Wnt/β-catenin signaling by apigenin appears not to be through its inhibition of CKII activity and other cellular targets in this pathway may be involved. Our data further suggested that apigenin could act on GSK3β *per se* or downstream of GSK3β in the Wnt/β-catenin signal transduction pathway. Apigenin has been shown to inhibit GSK3β kinase activity *in vitro* ^41^. Inhibition of GSK3β kinase activity in cells would result in the stability and accumulation of β-catenin. Given that apigenin can inhibit GSK3β kinase activity, β-catenin/TCF-mediated transcriptional activity would increase or at least maintain after co-treatment with LiCl and apigenin, as compared to treatment with LiCl alone. However, this is contradictory to our result (Fig. 4c). We observed that β-catenin/TCF-mediated transcriptional activity decreased concentration-dependently after co-treatment with LiCl and apigenin. Same inferences can be drawn from β-catenin/TCF-mediated transcriptional activity analyses of both Wnt-stimulated P19 cells and COS-7 cells treated with apigenin (Fig. 1a,b). These data suggested that apigenin appears not to inhibit the GSK3β kinase activity in our system.

We thus explored the effect of apigenin on the stabilization of β-catenin and found that the amount of total β-catenin decreased after apigenin treatment in Wnt-stimulated cells and Wnt-driven colorectal cancer cells (Figs 2c,d and 8). Furthermore apigenin treatment also reduced the cytoplasmic and nuclear β-catenin revealed by cell fractionation and immunofluorescent staining (Figs 5 and 6). In line with our findings, Shukla *et al*. also reported that apigenin reduced the levels of total β-catenin, cytoplasmic, and nuclear β-catenin in prostate cancer cells and TRAMP mice. However, the underlying mechanism of apigenin-induced β-catenin reduction was not touched^42^. In our study, we found that apigenin treatment induced the formation of autophagosomes, as revealed by the immunofluorescent staining of the autophagy marker LC3B (Fig. 7). We also observed the co-localization of β-catenin with LC3B in apigenin-treated cells (Fig. 7a). Besides, apigenin treatment led to the decrease in the level of β-catenin with a concomitant increase in LC3B II of HCT-116 cells (Fig. 8c). These results suggested that β-catenin was targeted to autophagosome probably by binding to LC3B and that β-catenin may be finally degraded by autophagy-lysosomal degradation system in apigenin-treated cells. Apigenin-induced down-regulation of β-catenin can be rescued by treatment with autophagy inhibitors include wortmannin and chloroquine, in Wnt-stimulated cells (Fig. 8a,b) and Wnt-active colorectal cancer cells (Fig. 8c,d), confirming the involvement of autophagy-lysosomal degradation system in the apigenin-induced down-regulation of β-catenin.

It is known that the levels of the autophagy markers, LC3B and p62/SQSTM1, will be increased during autophagy. Proteins destined for autophagy-lysosomal destruction will bind to the membrane-bound LC3B through the adaptor protein p62/SQSTM1 and then target proteins, LC3B, and p62 are all eventually degraded by lysosome^43^. As previously mentioned, we found that apigenin treatment led to the decrease in the level of β-catenin with a concomitant increase in LC3B II of HCT-116 cells (Fig. 8c), while the decrease in both levels of LC3B and/or p62 was observed by the same treatment in Wnt-stimulated P19 cells and WiDr cells (Fig. 8a,b,d). It is very likely that both the levels of LC3B and p62 may be transiently increased during autophagy, and however, the efficient autophagic flux outran the transient increase of both autophagy markers observed in Wnt-stimulated P19 cells and WiDr cells. Given that apigenin treatment increased the LC3B level in HCT-116 cells (Lane 2, Fig. 8c), it is expected that inhibition of autophagy by inhibitor wortmannin would lead to the decrease in LC3B level. However, co-treatment with wortmannin and apigenin further increased the LC3B level (Lane 3, Fig. 8c). The potential explanation for this result is that treatment with wortmannin will interfere with the formation of phagophore^44^, which in turn restrict the insertion of LC3B II into the phagophore membrane, probably leading to the defect in the degradation of LC3B with the protein cargos and hence the accumulation of LC3B. Petherick *et al*. have reported that the Wnt/β-catenin signaling pathway suppresses both basal and stress-induced autophagy^45^. Therefore it is reasoned that the levels of both autophagy markers LC3B and p62 should be reduced in the presence of Wnt. However, we observed the increase in the levels of both LC3B and p62 in Wnt-stimulated P19 cells as compared to that of control treatment (Fig. 8a,b). We suspected that the Wnt-induced LC3B and p62 may perform functions other than autophagy-lysosomal protein degradation, as the role of LC3B in vesicular transport has been proposed^46^. The other possibility is that this phenomenon may be due to different types of cells were used. Although the typical increase of both autophagy markers was not observed during apigenin-induced degradation of β-catenin, the levels of β-catenin were substantially rescued by treatment with autophagy inhibitors in our study (Fig. 8). Besides, our results showed that apigenin inhibited the phosphorylation of AKT, as well as p70 S6 kinase and 4E-BP-1, two known mTOR substrates (Fig. 9). These data suggested that the autophagy-lysosomal system is involved in apigenin-induced down-regulation of β-catenin through inhibition of the AKT/mTOR signaling pathway.

Wnt/β-catenin signaling pathway serves as a paradigm therapeutic target for the screening of chemical drugs, as this pathway is deregulated in a variety of diseases, including cancers^6^. Porcupine functions as a membrane-bound O-acyltransferase that palmitoleates the Wnt ligands for their efficient secretion in the Wnt signaling pathway. Porcupine inhibitors such as LGK974 can inhibit Wnt signaling by blocking Wnt secretion^47, 48^. Another chemical drug XAV939 can suppress the poly-ADP-ribosylation activity of tankyrase and thus inhibit the ubiquitination and degradation of Axin, and the accumulation of β-catenin^49, 50^. Besides, small molecules that inhibit Wnt signaling by interfering with the binding of Wnt ligand to its receptor Frizzled were identified^51^. These chemicals have been proved to target upstream of the Wnt signaling pathway, however, they are very likely fail to inhibit the intrinsic Wnt signaling generated from the mutations in components downstream of this pathway which include gene mutations in APC or β-catenin that lead to the aberrant accumulation of wild type or mutated β-catenin. Two strategies can be used to tackle with this problem. One is to erase these aberrantly accumulated β-catenin through cellular proteolytic machinery. The other is the functional inactivation of β-catenin by interfering with the following processes: (1) the mobilization of β-catenin into the nucleus; (2) the association of β-catenin with the transcription factor TCF/LEF, (3) the association of β-catenin with co-activators such as CBP; (4) the binding of β-catenin transcription activation complex onto the cognate promoter region, or (5) the events after complex binding onto the target DNA. Drugs that target on these processes have been identified. For example, a small molecule drug ICG-001 acts on the binding of β-catenin to co-activator CBP (cyclic AMP response element-binding protein)^52^. Resveratrol can disrupt the association of β-catenin with TCF^21^. Here we have shown that the harmless apigenin is able to drive the accumulated wild type (Fig. 8a,b,d) or mutant β-catenin to be degraded through the autophagy-lysosomal system (Fig. 8c), a way to eliminate aberrantly accumulated β-catenin caused by genetic mutations in Wnt signaling components. These results also indicated that the target site of apigenin situates downstream of the Wnt/β-catenin signaling pathway. Thus apigenin can be applied to the therapy of the diseases with either upstream or downstream intrinsic Wnt signaling. Further, combined treatment of apigenin with resveratrol or ICG-001 can be considered for more effective therapy as resveratrol or ICG-001 can functionally inactivate β-catenin escaped from the autophagy-lysosomal degradation triggered by apigenin.

In our studies, apigenin was shown to inhibit cell growth of Wnt-stimulated P19 cancer stem cells and Wnt-active WiDr, HCT-116, and SW480 colorectal cancer cells, with the IC50 to be 38.15 ± 1.32, 29.69 ± 0.15, 39.67 ± 0.99, 61.38 ± 2.29 μM, respectively (Fig. 3). We suspect that the observed different sensitivity of cancer cells to apigenin may be due to different genomic backgrounds such as gene mutations. SW480 cells have APC gene truncation and mutations in Ras and p53 genes. HCT-116 cells harbor mutations in Ras gene and β-catenin. WiDr (HT-29; ref. 53) cells possess APC gene truncation and mutation in p53^54^. The lack of Ras mutation in WiDr cells might explain why WiDr cells are more sensitive to apigenin treatment as compared to SW480 cells. For these cancer cells with higher IC50 for apigenin such as SW480, combined treatment of apigenin with other anti-cancer drugs may be a workable way for the therapy.

Cancer stem cells are a small subset of immortal cancer cells with stem cell properties such as its unlimited cell proliferation and ability to differentiate. They are believed to be responsible for cancer recurrence, metastasis, or resistance to radiotherapy and chemotherapy^55–59^. It is well-known that Wnt/β-catenin signaling is dispensable for the self-renewals and maintenance of cancer stem cells^60^. Therefore drugs that can inhibit Wnt/β-catenin signaling would be useful to eradicate cancer stem cells. P19 cells, the pluripotent embryonic carcinoma cells isolated from mouse, possess stem cell properties that can be differentiated into different cell types after stimulation with specific reagents^61, 62^ and thus can be used as a model cell line to study the effects of phytochemical drugs on cancer stem cells^63^. In this study, we found that the phytochemical apigenin is able to inhibit Wnt/β-catenin signaling in Wnt-stimulated P19 cells (Figs 1a and 2a,c) through the induction of autophagy-lysosomal degradation of β-catenin (Figs 7 and 8) and suppress the cell cycle progression and thus cell growth of Wnt-stimulated P19 cells (Fig. 3a and Supplementary Fig. S3), indicating the potential use of apigenin on the growth inhibition of cancer stem cells to prevent cancer relapse, metastasis, and drug resistance in the future.

**Figure 10:**
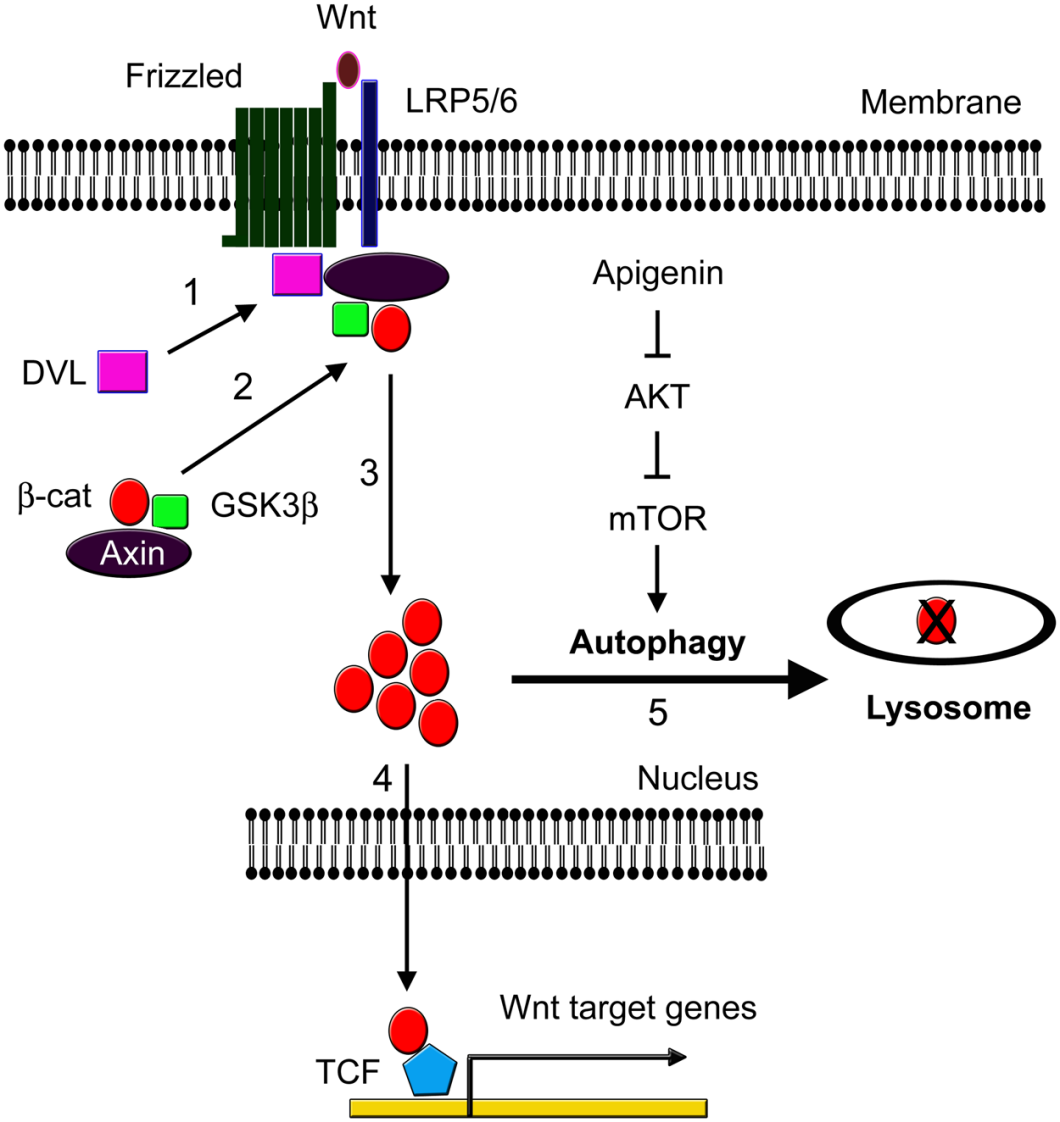
A model depicting the involvement of autophagy-lysosomal system in apigenin-induced degradation of β-catenin during Wnt signaling. In the presence of Wnt, Wnt ligand binds to its cognate receptor Frizzled and co-receptor LRP5/6. Dishevelled (DVL) moves from the cytoplasm to the plasma membrane and associates with Frizzled (step 1). Axin complex, including β-catenin and GSK3β, will also be translocated to the cell membrane and bind to phosphorylated LRP5/6 through Axin (step 2). β-catenin is stabilized and accumulated in the cytoplasm (step 3), and then enters the nucleus to associate with TCF/LEF and activates the expression of Wnt-responsive genes (step 4). Apigenin treatment suppresses the AKT/mTOR signaling pathway and induces the sequestration and degradation of β-catenin inside the lysosome (step 5), contributing to down-regulation of β-catenin during Wnt signaling.

Here, we propose a model to depict the action mechanism of apigenin on Wnt/β-catenin signaling according to our studies. As shown in Fig. 10, binding of Wnt to receptor Frizzled and co-receptor LRP5/6 activates the mobilization of DVL onto the cell membrane to associate with Frizzled (step 1). Axin complex including GSK3β and β-catenin will be translocated to the cell membrane and interact with LRP5/6 through Axin (step 2). β-catenin is then stabilized and accumulated in the cytoplasm (step 3). The stabilized β-catenin enters the nucleus and complexes with TCF/LEF proteins to activate the expression of Wnt target genes (step 4). Apigenin suppresses the AKT/mTOR signaling pathway and impels the incorporation of β-catenin, no matter wild-type or mutant form, into autophagosomes with the help of LC3 and p62. Autophagosomes are then fused with lysosome to form autolysosome, inside which β-catenin, LC3B, and p62 are finally degraded (step 5), releasing digested materials for anabolic reactions. In conclusion, we have uncovered that, during Wnt signaling, apigenin can steer dis-regulated β-catenin to autolysosome for degradation, a way to diminish the unusual accumulated β-catenin and thus reduce the β-catenin-mediated downstream Wnt signaling. Our findings also suggested that any reagents or strategies that can drive β-catenin for autolysosomal degradation will be promising for the therapy of Wnt-related diseases including colorectal cancers.

## Methods

### Reagents

Apigenin was obtained from Sigma-Aldrich (St. Louis, MO, USA) and dissolved in dimethyl sulfoxide (DMSO) at the concentration of 50 mM. Chloroquine and wortmannin was obtained from Sigma-Aldrich and dissolved in sterile water and DMSO respectively. 3-(4,5-Dimethyl-2-thiazolyl)-2,5-diphenyl-2H-tetrazolium bromide (MTT) was purchased from Sigma-Aldrich and prepared in phosphate-buffered saline. Paraformaldehyde was purchased from Electron Microscopy Sciences (Hatfield, PA, USA). 4′,6-diamidino-2-phenylindole (DAPI) was from Invitrogen (Camarillo, CA, USA). Dual-Luciferase Reporter Assay System was from Promega (Madison, WI, USA). Complete™ Protease Inhibitor Cocktail Tablet, EDTA-free, and PhosSTOP Phosphatase Inhibitor Cocktail Tablets were from Roche (Mannheim, Germany). Triton X-100 was from Sigma-Aldrich. SuperScript III First-Strand Synthesis SuperMix and Platinum PCR SuperMix were from Invitrogen. Primary antibodies for β-tubulin (Sigma-Aldrich, St. Louis, MO, USA), GAPDH, brachyury (T), Axin2 (Conductin), c-Myc (9E10), lamin A, SQSTM1/p62 (Santa Cruz Biotechnology, Santa Cruz, CA, USA), β-catenin (BD Transduction Laboratories, San Jose, CA, USA), LC3B, cyclin D1, 4E-BP1 (53H11), phospho-4E-BP1 (Ser65), p70 S6 kinase, phospho-p70 S6 kinase (Thr389), pan Akt (C67E7), and phospho-Akt (Ser473) (D9E) (Cell Signaling, Danvers, MA, USA) were obtained from the indicated vendors. The human colorectal cancer cell lines HCT-116 (BCRC 60349), SW480 (BCRC 60249), WiDr (BCRC 60157) cells, the mouse embryonal carcinoma P19 cells (BCRC 60052) and the African green monkey fibroblast COS-7 cells (BCRC 60094) were obtained from Bioresource Collection and Research Center (BCRC), Hsinchu, Taiwan. 3X-Flag-tagged human DVL2 cDNA was from Dr. Jeff Wrana (Addgene plasmid number: 24802). All other chemicals were from either Merck or Sigma unless specified. pLRP5ΔN was constructed by inserting the PCR-amplified cDNA fragment containing LRP5 transmembrane and C-terminal region into the pcDNA3-HA(3) plasmid, a pcDNA-based vector with an engineered HA tag, and the insert was completely sequenced to confirm its identity.

### Cell culture and transfection

HCT-116, SW480, WiDr, and COS-7 cells were cultured in Dulbecco’s Modified Eagle’s medium (DMEM) supplemented with 10% of fetal bovine serum (FBS, Gibco, Life Technologies), 1% of penicillin and streptomycin (Gibco), and sodium pyruvate. P19 cells were cultured in Minimum Essential Medium (MEM) alpha with 2.5% of fetal bovine serum (Hyclone), 7.5% of bovine calf serum (Hyclone), 1% of penicillin and streptomycin, and sodium pyruvate. All cells were incubated in a 37 °C humidified chamber with 5% of CO_2_. The culture of L cells and L-Wnt-3a cells and preparation of control and Wnt-3a conditioned media were as described previously^7, 21^. Cells were transfected with Lipofectamine 2000 (Life Technologies) according to the company’s brochure.

### Cell viability assay

We used MTT assay to measure the cell viability^21^. Cells were seeded at the density of 2 × 10^3^ cells per well in a 96-well dish and kept in the culture chamber for overnight to allow the cells to attach. Cells were treated with different concentrations of apigenin in the culture medium or Wnt-conditioned medium (for P19 cells) and cultured for additional four days. Cultured medium was then removed and 50 μL of MTT solution (2 mg/mL) was added into the cells carefully and the dish was placed in the culture chamber for additional 4 h. At the end of incubation, the dish was spun at 2,500 rpm for 20 min at 4 °C. MTT solution was carefully removed and 100 μL of DMSO was pipetted into each well to dissolve the developed formazan crystals. After 20 min of incubation, the dish was read at 570 nm in an ELISA reader (Multiskan Ex, Thermo Electron Corporation) and the data was further processed by Excel to calculate the relative cell viability.

### Cell fractionation

Cells were treated as described in the text and fractionation of cells into nuclear and cytosolic fractions was performed by using the NE-PER Nuclear and Cytoplasmic Extraction Reagents (Thermo Scientific) according to the procedures provided by the company.

### Immunofluorescent staining

Cells were seeded on coverslips, treated with drugs, and fixed with 4% of paraformaldehyde (PFA) for 15 min at room temperature. After three washes in PBS for 10 min, cells were permeabolized with 0.1% of Triton X-100 in PBS and washed with PBS for three times. For the detection of autophagosomes, cells were further permeabolized with cold methanol at −20 °C for 10 min after PFA fixation but omit the Triton X-100 step. Cells were then blocked in 10% of normal goat serum (NGS, Gibco, Invitrogen) in PBS for 30 min at ambient temperature before the incubation with primary antibodies in 1.0% of NGS in PBS at 4 °C for overnight. On the next day, cells were warmed up for 10 min at room temperature and washed three times with PBS for 10 min each time. Cells were then incubated with secondary antibody Alexa Flor 488 goat anti-mouse IgG, Alexa Flor 488 goat anti-rabbit IgG, or Alexa Flor 546 goat anti-mouse IgG (Molecular Probes; 1:500) for 1 h at room temperature. To prevent the quench from light, cells were always kept in the dark after the step with secondary antibodies. After incubation with DAPI (1:1500 dilution in PBS; 5 mg/ml in DMSO) for 10 min, cells were washed three more times with PBS, mounted in 50% of glycerol in PBS, and sealed upside-down onto the slide with nail polish covered around the coverslip. Images were recorded by using a Zeiss AXIO Observer. D1 Fluorescence microscope equipped with an imaging system, AxioCam MRm (Carl Zeiss, Germany).

### Dual luciferase activity assay

P19, COS-7, HCT-116 or SW480 cells (1 × 10^5^/well) were seeded onto a 24-well dish and incubated for overnight. For HCT-116 and SW480 cells, cells were transfected with the Wnt reporter pGL3-OT and the normalization vector pTK-Renilla (dual reporters) and treated with different concentrations of apigenin before the dual luciferase activity assay. To test the effects of apigenin on upstream or downstream of DVL and LRP5, P19 cells were transfected with dual reporters, the effector plasmid (DVL2 and pLRP5ΔN) or empty vector, and treated with different concentrations of apigenin for the indicated time. To test the effects of apigenin on Wnt signaling, P19 cells or COS-7 cells were transfected with dual reporters, and either treated with different concentrations of apigenin in Wnt-3a-conditioned medium or incubated in control-conditioned medium. For LiCl treatment, Wnt-reporter-transfected P19 cells were incubated in culture medium, treated with LiCl in culture medium, or treated with LiCl and different concentrations of apigenin in culture medium before the dual luciferase activity assay. Each treatment was in three replicates. Cells were then washed once with PBS and lysed in 1X passive lysis buffer. Cell lysates were collected and spun at 12,000 rpm for 1 min. Supernatant was transferred into a new eppendorf tube and kept at −80 °C or proceeded to the measurement of dual luciferase activities using the Dual Luciferase Reporter Assay System on a GLOMAX 20/20 luminometer (Turner BioSystems, Sunnyvale, CA). Firefly luciferase activity was normalized by Renilla luciferase activity^7^.

### RT-PCR analysis

P19 cells or HCT-116 cells were treated with different concentration of apigenin or carrier reagent (DMSO) in culture medium (HCT-116 cells) or in Wnt-3a-conditioned media (P19 cells) for the indicated time. Total RNAs were isolated from treated cells by using the Trizol reagent (Invitrogen) according to the company’s protocol. 5 μg of RNAs were used for the synthesis of cDNAs by using a cDNA synthesis kit (Invitrogen) and the resulting cDNAs were used as the template for the amplification of Wnt target genes or *GAPDH* gene by using the PCR SuperMix (Invitrogen). Gene-specific primers used were: mouse *Axin2* (amplicon, 237 bp), forward, 5′-GTTAGTGACTCTCCTTCCAGATCC-3′, reverse, 5′-GAGTGTAAAGACTTGGTCCACCTG-3′, human *Axin2* (amplicon, 237 bp), forward, 5′-GTTGGTGACTTGCCTCCCGGACCC-3′, reverse, 5′-GAGTGTAAGGACTTGGTCCACCGG-3′, human and mouse *GAPDH* (amplicon, 539 bp), forward, 5′-CGTATTGGGCGCCTGGTCACC-3′, reverse, 5′-GAGGGGCCATCCACAGTCTTC-3′, human *c*-*Myc* (amplicon, 505 bp), forward, 5′-CCAGGACTGTATGTGGAGCGG-3′, reverse, 5′-CTTGAGGACCAGTGGGCTGTG-3′, mouse *T* (amplicon, 371 bp), forward, 5′-GTGACCAAGAACGGCAGGAGGATG-3′, reverse, 5′-AAGCAGTGGCTGGTGATCATGCG-3′, and human and mouse *cyclin D1* (amplicon, 257 bp), forward, 5′-ATGGAACACCAGCTCCTGTGCTGCG-3′, reverse, 5′-TCCAGGTAGTTCATGGCCAGCGGG-3′.

### Western blotting analysis

Treated cells were collected, resuspended in protein lysis buffer (50 mM Tris-HCl, pH7.4, 150 mM NaCl, 5 mM EDTA, 1% [v/v] of Triton X-100) supplemented with the protease inhibitor and phosphatase inhibitor (Roche), and incubated for 10 min at 4 °C with gentle shaking. After centrifugation at 12,000 rpm for 15 min at 4 °C, clear cell lysate was transferred to a new tube. Protein concentration was determined by the Bradford method using Protein Assay Dye Reagent (Bio-Rad, Hercules, CA, USA). Equal amount of cell lysate was resolved on a SDS-PAGE gel and transferred onto PVDF membrane (Immobilion-P transfer membrane, Millipore, Billerica, Ma, USA). The transblot was incubated in the blocking solution (5% skim milk in TBS, 25 mM Tris, 150 mM NaCl, pH 7.4) for 1 h at room temperature. After a 10-minute incubation in TBS containing 1% skim milk, the blot was then incubated in primary antibody for 2 h at room temperature or overnight at 4 °C on a rotatory shaker. The blot was washed three times in TBST (0.1% [v/v] Tween-20 in TBS) for 10 min each time, and soaked in HRP-coupled goat anti-mouse or rabbit secondary antibodies for 1 h at room temperature. After three additional washes in TBST and one wash in TBS, the blot was layered with ECL reagent (Immobilion Western chemiluminescent HRP substrate, Millipore) and exposed to X-ray film (Konica Minolta, Tokyo, Japan) to reveal the signals.

## Electronic supplementary material

Supplementary Information
