# Supplementary material for: Apigenin-induced lysosomal degradation of β-catenin in Wnt/β-catenin signaling
Source: Sci Rep. 2017 Mar 23;7:372. doi: 10.1038/s41598-017-00409-z (PMC5428476; doi:10.1038/s41598-017-00409-z)
Supplement: Supplementary file 1 — Supplementary Information [file 41598_2017_409_MOESM1_ESM.pdf]

Apigenin-induced lysosomal degradation of β-catenin in Wnt/β-catenin signaling

Chung-Ming Lin[1], Hsin-Han Chen[2], Chun-An Lin[3], Hui-Chung Wu[1], Jim Jinn-Chyuan Sheu[4], Hui-Jye Chen[3, 5, 6,*]

[1]Department of Biotechnology, Ming-Chuan University, Taoyuan 33348, Taiwan

[2]Division of Plastic and Reconstructive Surgery, Department of Surgery, China Medical University Hospital, Taichung 40402, Taiwan

[3]Graduate Institute of Basic Medical Science, China Medical University, Taichung 40402, Taiwan

[4]Institute of Biomedical Sciences, National Sun Yat-sen University, Kaohsiung 80424, Taiwan

[5]Department of Nursing, Asia University, Taichung 40354, Taiwan

[6]Graduate Institute of Biomedical Sciences, China Medical University, Taichung 40402, Taiwan

*Corresponding author. E-mail: huijyechen@mail.cmu.edu.tw

Supplementary Data

Supplementary Figures

Supplementary Figure 1

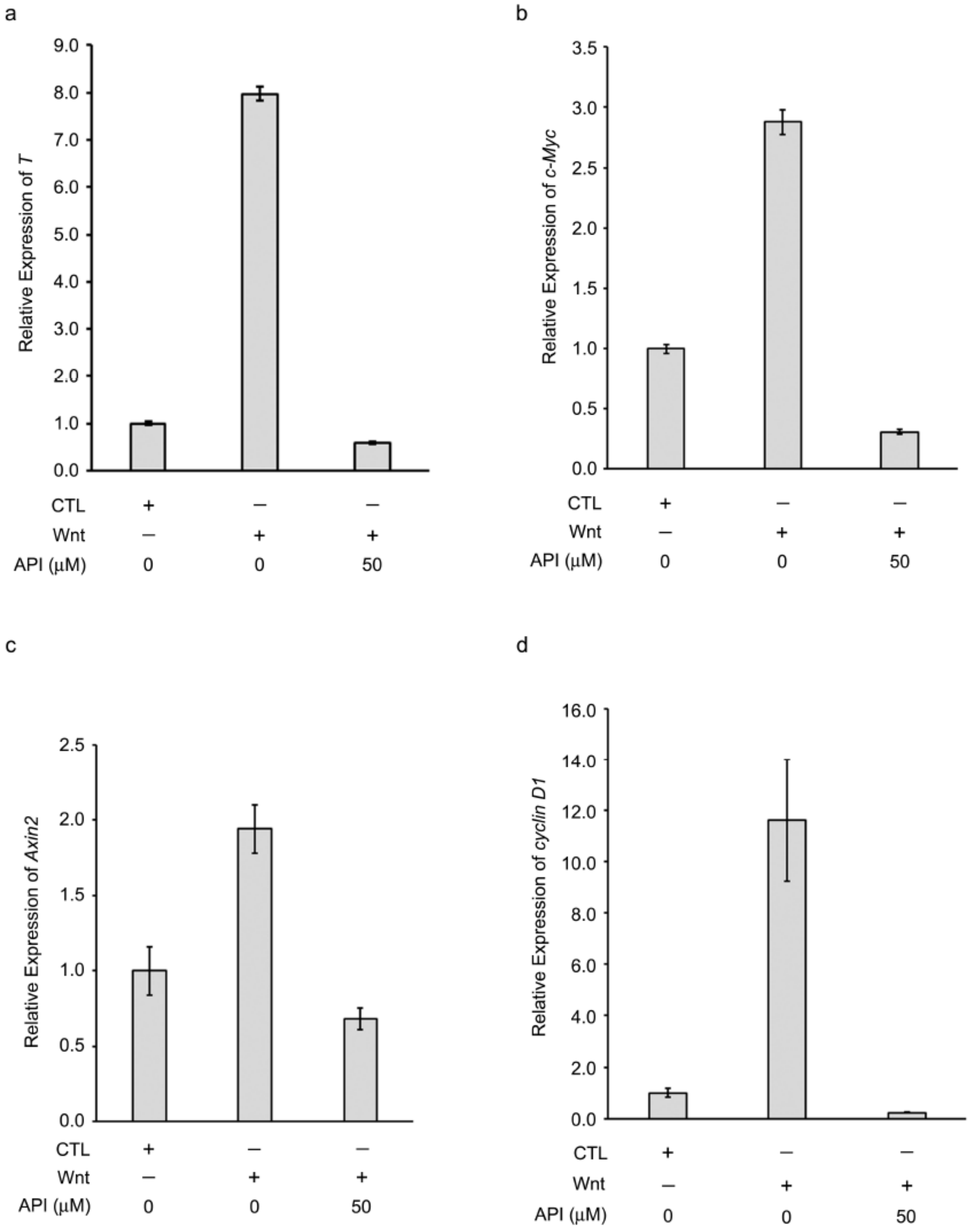

Supplementary Figure S1. Apigenin suppresses the mRNA expression of Wnt target genes in Wnt-stimulated P19 cells. Cells were treated with control-conditioned medium (CTL), Wnt-3a-conditioned medium (Wnt), or 50 μM of apigenin in

Wnt-3a-conditioned medium for 16 h, and the expression of (a) *T*, (b) *c-Myc*, (c) *Axin2*, and (d) *cyclin D1* was examined by quantitative real-time PCR analyses. The expression of Wnt target gene in cells treated with control-conditioned medium was set as 1.0 and the expression of Wnt target gene in cells treated with Wnt-3a-conditioned medium or in drug-treated cells with Wnt-3a-conditioned medium were calculated accordingly. Data were mean±SEM calculated from three replicates.

Supplementary Figure 2

a

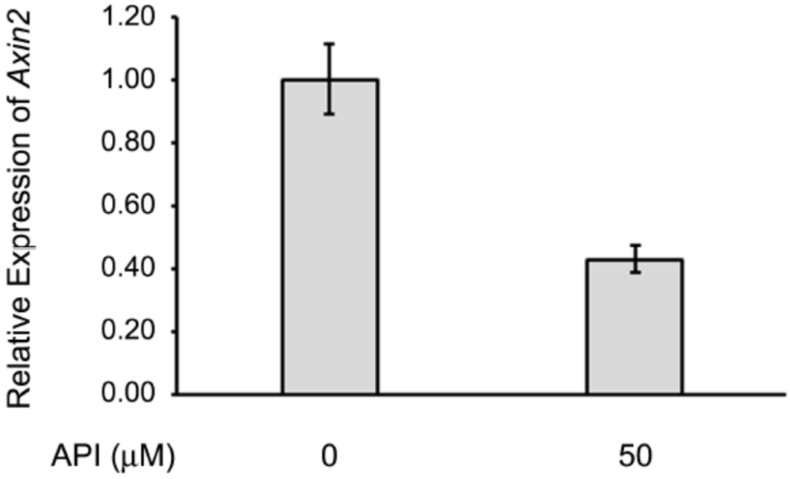
Relative Expression of *Axin2*
1.20
1.00
0.80
0.60
0.40
0.20
0.00
API (μM)
0
50

b

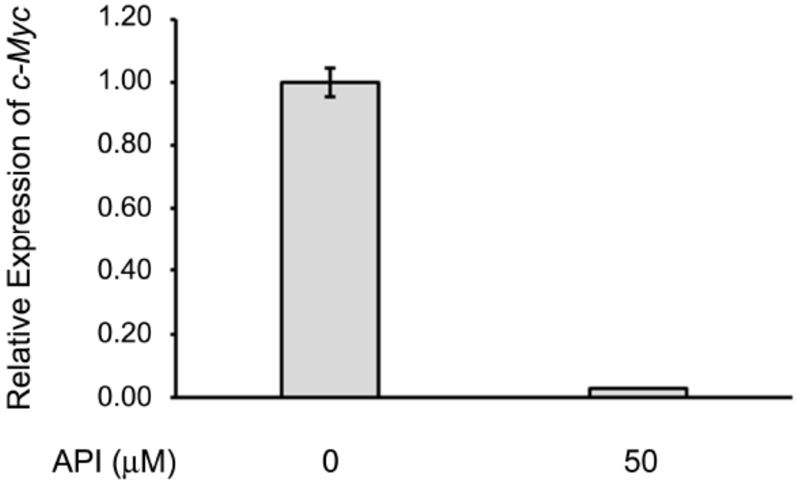
Relative Expression of *c-Myc*
1.20
1.00
0.80
0.60
0.40
0.20
0.00
API (μM)
0
50

c

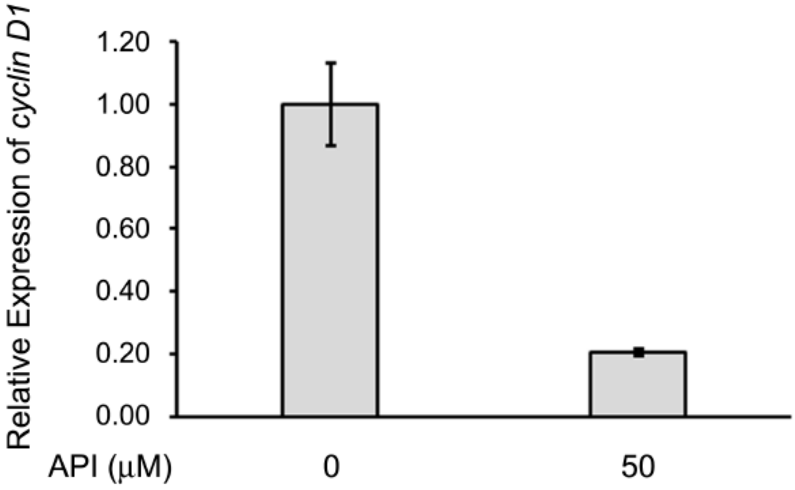
Relative Expression of *cyclin D1*
1.20
1.00
0.80
0.60
0.40
0.20
0.00
API (μM)
0
50

Supplementary Figure S2. Apigenin decreases the mRNA expression of Wnt target genes in HCT-116 cells. Cells were treated with 50 μM of apigenin or carrier reagent (DMSO) for 20 h, and the expression of (a) *Axin2*, (b) *c-Myc*, and (c) *cyclin D1* was examined by quantitative real-time PCR analyses. The expression of Wnt target gene without drug treatment was set as 1.0 and the expression of Wnt target gene in apigenin-treated cells were calculated accordingly. Data were mean±SEM calculated from three replicates.

Supplementary Figure 3

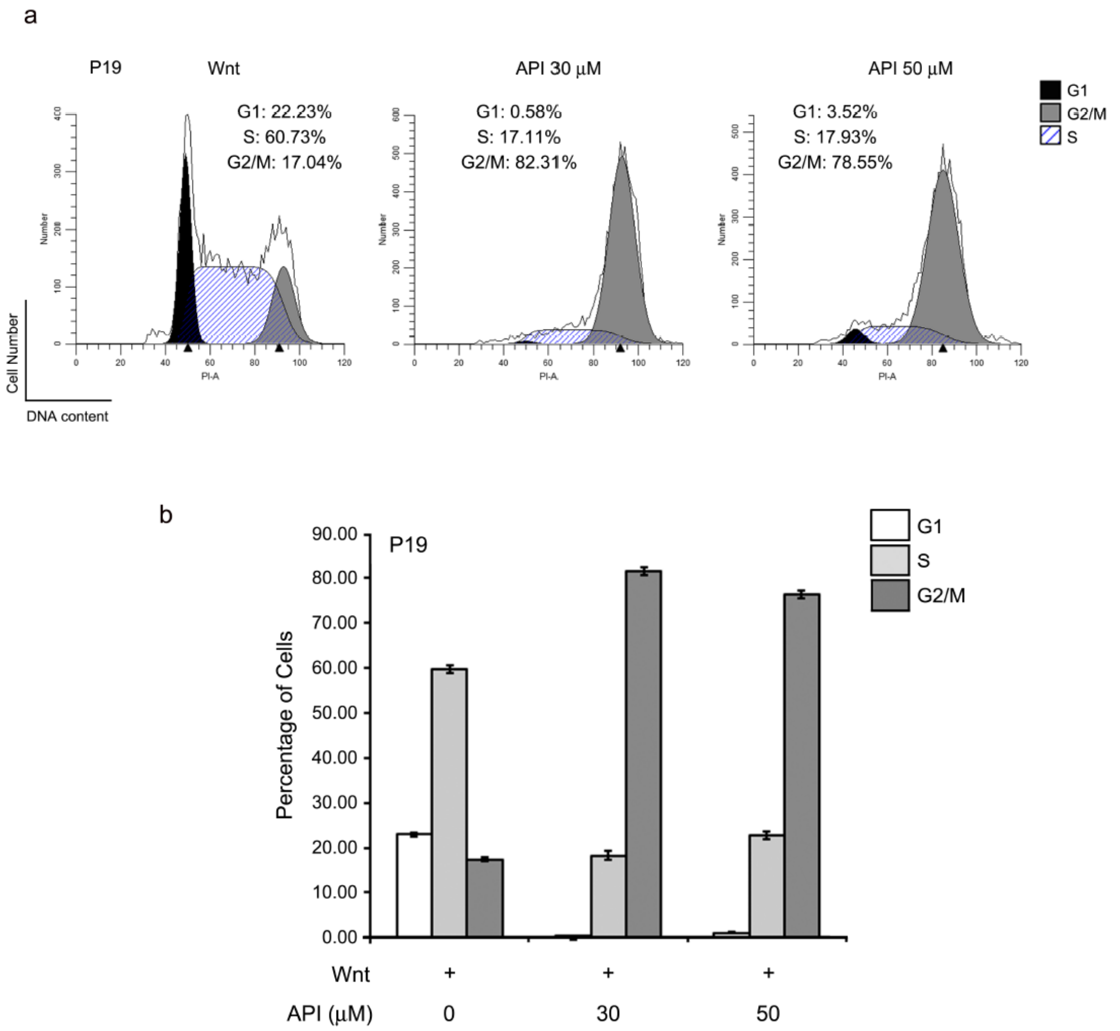

Supplementary Figure S3. Apigenin changes the cell cycle distribution of Wnt-stimulated P19 cells. Cells were treated with Wnt-3a-conditioned medium (Wnt),

or different concentrations of apigenin (0 μM, 30 μM, and 50 μM) in Wnt-3a-conditioned medium for 16 h, then fixed and stained with propidium iodide, and analyzed by flow cytometry. (a) The number of cells in each phase of the cell cycle was expressed as the percentage of gated events. (b) Plots that demonstrate percentage of cells in G1, S, and G2/M phases and represent the means±SD of three replicates.

Supplementary Figure 4

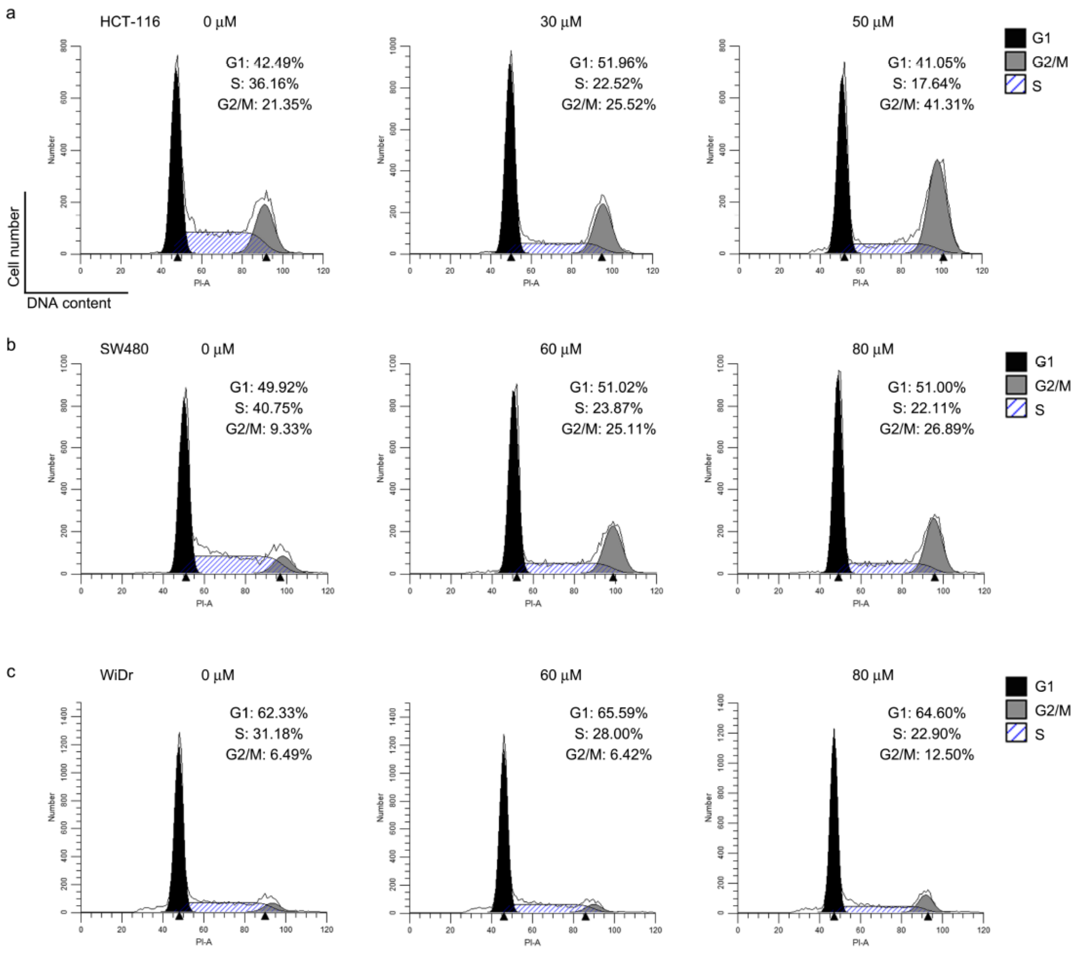

d

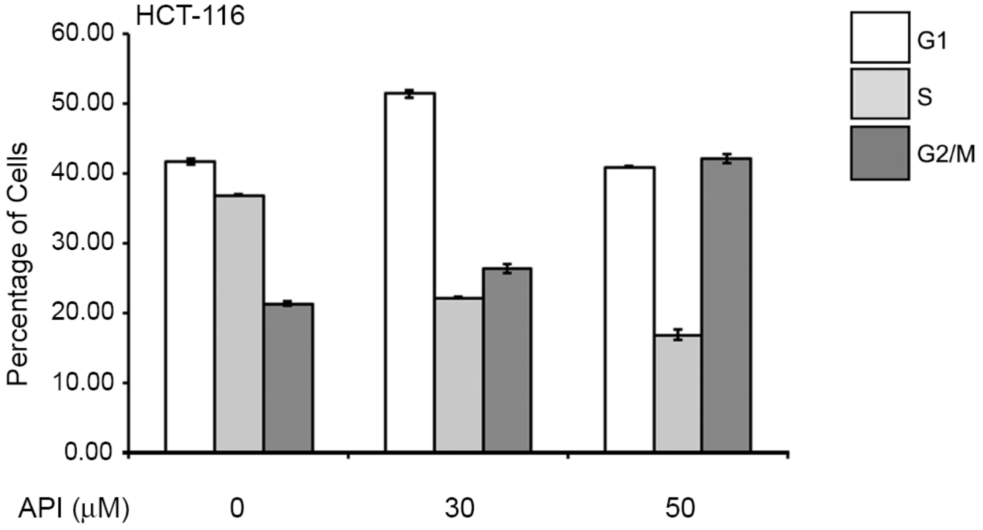
HCT-116
Percentage of Cells
60.00
50.00
40.00
30.00
20.00
10.00
0.00
API (μM)
0
30
50
G1
S
G2/M

e

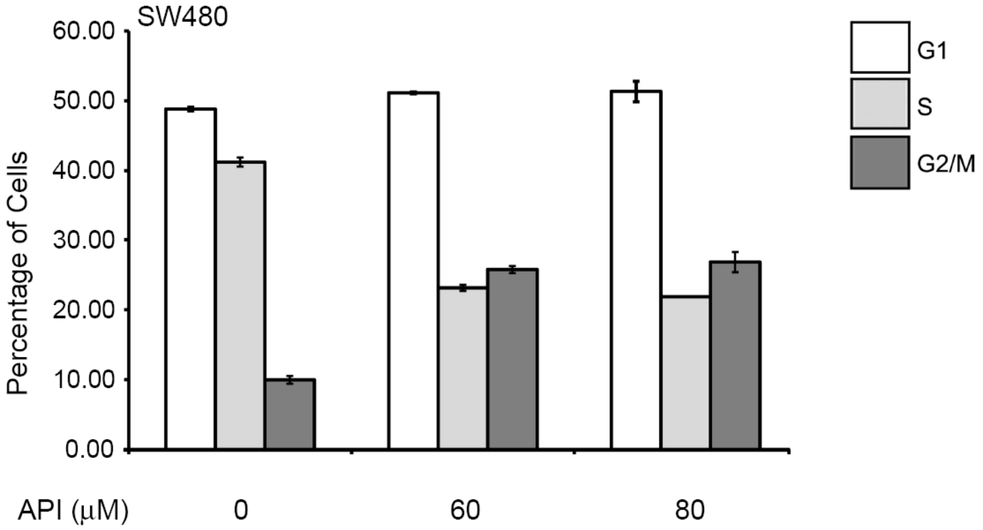
SW480
Percentage of Cells
60.00
50.00
40.00
30.00
20.00
10.00
0.00
API (μM)
0
60
80
G1
S
G2/M

f

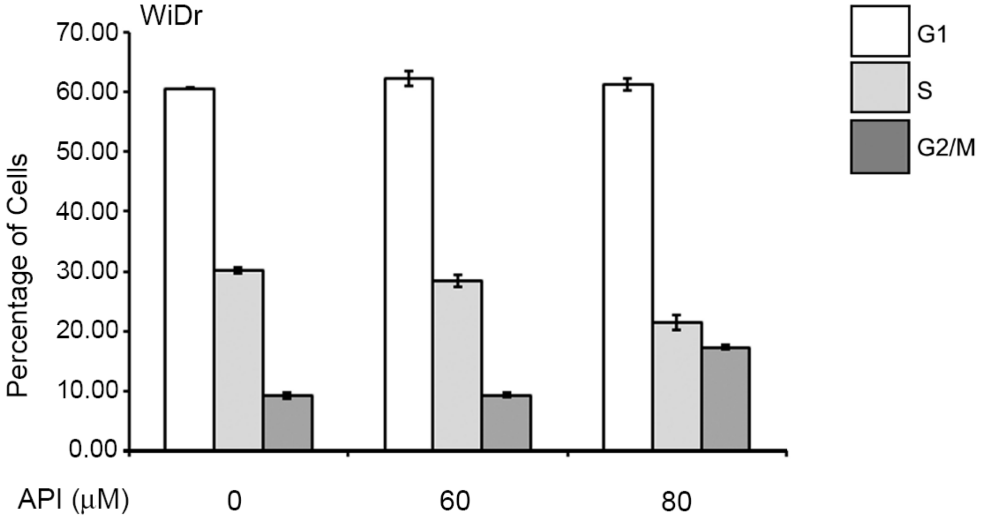
WiDr
Percentage of Cells
70.00
60.00
50.00
40.00
30.00
20.00
10.00
0.00
API (μM)
0
60
80
G1
S
G2/M

Supplementary Figure S4. Apigenin re-distributes the cell cycle of HCT-116, SW480, and WiDr colorectal cancer cells. Cells were treated with different concentrations of apigenin (a, 0, 30, and 50 μM for HCT-116 cells; b and c, 0, 60, and 80 μM for SW480 and WiDr cells) in complete DMEM medium for 20 h, fixed and stained with propidium iodide, and cell cycle distribution was analyzed by flow cytometry. (a-c)The number of cells in each phase of the cell cycle was expressed as the percentage of gated events. (d-f) Plots that demonstrate percentage of cells in G1, S, and G2/M phases and reported as the means±SD of three replicates.

Supplementary Methods

Quantitative real-time PCR (qRT-PCR) analysis. RNA isolation and cDNA synthesis were described previously in methods of the main text. The resulting cDNAs were used for subsequent real-time PCR analyses. The reaction mixture containing 250 nM of gene-specific forward and reverse primers, 1X KAPA SYBR FAST qPCR Master Mix (KAPA Biosystems, Boston, MA, USA), and cDNAs were mixed in a 0.1 mL tube. Real-time PCR analyses were performed in the CFX96 Real-Time System (Bio-Rad Laboratories, Singapore) and analyzed in the Bio-Rad CFX manager software. Successful amplification was revealed by examining the respective melting curve and confirmed by examining the PCR products in agarose gel electrophoresis.

Mouse primers for qRT-PCR. Primers for qRT-PCR analyses of mouse genes including *Brachyury* (*T;* amplicon, 79 bp), *c-Myc* (amplicon, 150 bp), *Axin2* (amplicon, 296 bp), *cyclin D1* (amplicon, 117 bp), and *GAPDH* (amplicon, 109 bp): *Brachyury* (*T*) forward primer: 5′-AACAGCTCTCCAACCTAT-3′; *Brachyury* (*T*) reverse primer: 5′-CGAGGCTAGACCAGTTAT-3′; *c-Myc* forward primer:

5′-CGCGATCAGCTCTCCTGAAA-3′; *c-Myc* reverse primer: 5′-GCTGTACGGAGTCGTAGTCG-3′; *Axin2* forward primer: 5′-GAGTAGCGCCGTGTTAGTGACT-3′; *Axin2* reverse primer: 5′-CCAGGAAAGTCCGGAAGAGGTATG-3′; *cyclin D1* forward primer: 5’-GTTCATTTCCAACCCACCCTC-3’; *cyclin D1* reverse primer: 5′-AGAAAGTGCGTTGTGCGGTAG-3′; *GAPDH* forward primer: 5′-CGACTTCAACAGCAACTC-3′; *GAPDH* reverse primer: 5′-GTAGCCGTATTCATTGTCAT-3′. The annealing temperatures used for amplification were between 50 ^{o}C and 60 ^{o}C.

Human primers for qRT-PCR. Primers for qRT-PCR analyses of human genes including *Axin2* (amplicon, 232 bp), *c-Myc* (amplicon, 75 bp), *cyclin D1* (amplicon, 135 bp), and *GAPDH* (amplicon, 113 bp): *Axin2* forward primer: 5′-GCAGACGACGAAGCATGTC-3′; *Axin2* reverse primer: 5′-GCCTTTCCCATTGCGTTTGG-3′; *c-Myc* forward primer: 5′-TAGTGGAAAACCAGCAGCCT-3′; *c-Myc* reverse primer: 5′-AGTCGAGGTCATAGTTCCTGTTG-3′; *cyclin D1* forward primer: 5′-GCTGCGAAGTGGAAACCATC-3′; *cyclin D1* reverse primer: 5′-CCTCCTTCTGCACACATTTGAA-3; *GAPDH* forward primer: 5′-CATGAGAAGTATGACAACAGCCT-3′; *GAPDH* reverse primer: 5′-AGTCCTTCCACGATACCAAAGT-3′. The annealing temperatures used for amplification were between 50 ^{o}C and 60 ^{o}C.

Cell cycle analyses. Cells were treated with different concentrations of apigenin for the indicated time, and all cells were collected after trypsinization, washed two times with PBS, counted and suspended at 2 X 10^{6} cells per mL in iced-cold PBS. 2 X 10^{6}

of cells were then collected after a spin at 1000 rpm for 10 min at 4 ^{o}C and suspended in 1.5 mL of iced-cold PBS. 3.5 mL of iced-cold absolute ethanol were slowly and drop-wisely added into the 1.5 mL of cells with gentle vortexing. Fixed cells were stored at -20 ^{o}C overnight. On the next day, cells were washed twice with iced-cold 2% BSA/PBS, resuspended in staining solution (2% BSA/PBS containing 0.1% Triton X-100, 20 μg/mL of propidium iodide (Sigma-Aldrich, St. Louis, MO, USA) and 200 μg/mL of RNase A (Invitrogen, Camarillo, CA, USA)), and incubated at 37 ^{o}C for 30 min. Data were acquired with a flow cytometer (BD FACSCanto) and ModFit LT (version 4.1) is used to fit the obtained data to various cell cycle models.
